# Synthesis and Preliminary Biological Evaluation of 1,3,5-Triazine Amino Acid Derivatives to Study Their MAO Inhibitors

**DOI:** 10.3390/molecules200915976

**Published:** 2015-09-02

**Authors:** Sherine N. Khattab, Hosam H. Khalil, Adnan A. Bekhit, Mohamed Mokbel Abd El-Rahman, Ayman El-Faham, Fernando Albericio

**Affiliations:** 1 Department of Chemistry, Faculty of Science, Alexandria University, P. O. Box 426, Ibrahimia, Alexandria 21321, Egypt; E-Mails: sh.n.khattab@gmail.com (S.N.K.); chemhosam1@yahoo.com (H.H.K.); mohamedmokbel80@gmail.com (M.M.A.E.-R.); 2 Department of Pharmaceutical Chemistry, Faculty of Pharmacy, Alexandria University, Alexandria 21521, Egypt; E-Mail: adnbekhit@hotmail.com; 3 Department of Chemistry, College of Science, King Saud University, P. O. Box 2455, Riyadh 11451, Saudi Arabia; 4 Catalysis and Peptide Research Unit, School of Health Sciences, University of KwaZulu-Natal, Durban 4001, South Africa; 5 School of Chemistry and Physics, University of KwaZulu-Natal, Durban 4001, South Africa; 6 Institute for Research in Biomedicine and CIBER-BBN, Barcelona 08028, Spain; 7 Department of Organic Chemistry, University of Barcelona, Barcelona 08028, Spain

**Keywords:** 1,3,5-triazine derivatives, amino acids, morpholine, piperidine, monoamine oxidase

## Abstract

Three series of 4,6-dimethoxy-, 4,6-dipiperidino- and 4,6-dimorpholino-1,3,5-triazin-2-yl) amino acid derivatives were synthesized and characterized. A preliminary study for their monoamine oxidase inhibitory activity showed that compounds **7**, **18**, and **25** had MAO-A inhibition activity comparable to that of the standard clorgyline, with apparently more selective inhibitory activity toward MAO-A than MAO-B and no significant acute toxicity.

## 1. Introduction

Human monoamine oxidases A and B (MAO-A and B) are the most widely studied flavin-dependent amine oxidases. They are located in the mitochondrial outer membranes of neuronal, glial, and other cells particularly abundant in the liver and brain [1,2]. These FAD-dependent enzymes catalyze the oxidative deamination of several endogenous and exogenous monoamines and are responsible for the regulation and metabolism of major monoamine neurotransmitters, such as serotonin (5-OH tryptamine), noradrenaline, and dopamine [1,2,3]. The two mammalian isoforms of these enzymes are characterized by their distinct sensitivity to inhibitors and specificity to substrates. Thus, MAO-A is selectively inhibited by clorgyline and preferentially metabolizes serotonin: whereas MAO-B is inhibited by l-deprenyl and preferentially metabolizes benzylamine and phenylethylamine as substrates [4]. Among selective MAO inhibitors, those against MAO-A are used as anti-depressant and anti-anxiety drugs and have been claimed to protect neuronal cells against apoptosis [5,6]. In contrast, MAO-B inhibitors have been found to be beneficial in the treatment of Parkinson’s disease and Alzheimer’s disease. Early MAO-inhibitors introduced into clinical practice for the treatment of depression were abandoned due to adverse side effects, such as the “cheese effect”, which is characterized by hypertensive crises [4], and because the mechanism of interaction of several new drugs with MAOs has not been yet fully characterized. For these reasons, research has been aimed at the synthesis of new potential agents with clinical applications.

Recently, we have demonstrated a series of 3-benzyl-2-substituted quinoxalines as selective MAO-A inhibitors bearing substituted amino or hydrazino functionalities at position 2 [7] and novel structural variants of [1,2,4]triazolo[4,3-*a*]quinoxaline derivatives [8]. In addition, substituted pyridazine-1-yl acetic acid derivatives [9], and α-ketoamino acid ester derivatives [10] were established as selective monoamine oxidase-A inhibitors.

1,3,5-triazine derivatives are an important class of small molecules with anti-cancer [11,12,13,14,15,16] and anti-viral activity, among others [17]. These compounds are known to be VLA-4 integrin antagonists, anti-inflammatory agents [18], sorbitol dehydrogenase inhibitors [19], estrogen receptor modulators [20], potential anti-trypanosomal drugs [21], antimalarial agents [22,23,24,25,26,27,28,29], hypolipidemic agents, [30] and antimicrobial agents [31,32,33].

Here, we prepared three small libraries of molecules based on amino acid-substituted 1,3,5-triazine and evaluated their capacity to inhibit MOAs.

**Figure 1:**
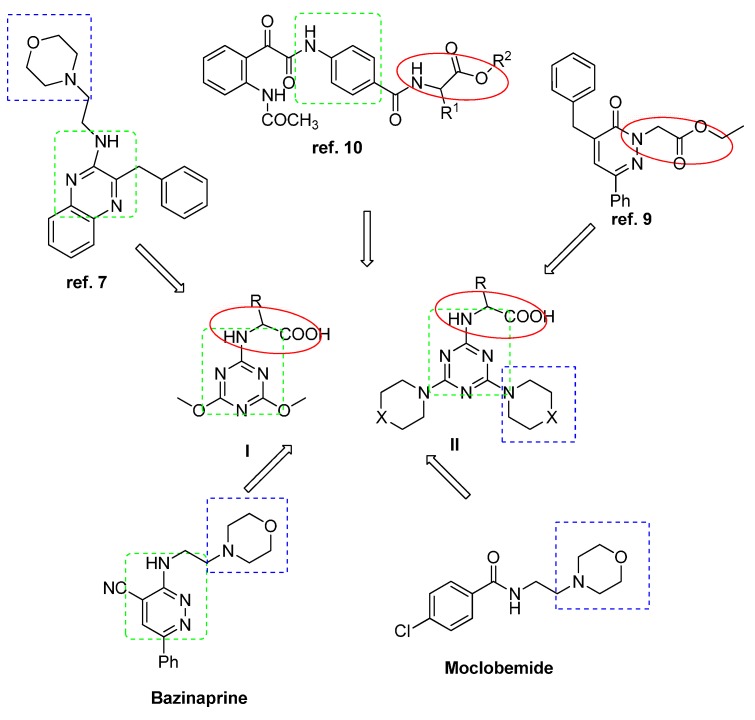
Planned modification and newly designed MAO inhibitors. I = (4,6-dimethoxy-1,3,5-triazin-2-yl) amino acid derivatives; X = O; II is (4,6-dimorpholino-1,3,5-triazin-2-yl) amino acid derivatives or X = CH_2_; II is (4,6-dipiperidino-1,3,5-triazin-2-yl) amino acid derivatives.

The aim of the present study was to tailor MAO-A inhibitors by designing a hybrid from different possible active sites of previously known MAO-A inhibitors, based on the following considerations: (i) the presence of electron-rich aromatic moieties (e.g., moclobemide [34],bazinaprine [35], quinoxaline derivatives [7,8]); (ii) the presence of morpholine moiety (e.g., moclobemide [34], bazinaprine [35]); and (iii) the presence of amino acid moiety [9,10]). The target compounds were designed to study the effect of molecular variation on MAO inhibitory activity, Figure 1.

## 2. Results and Discussion

### 2.1. Chemistry

We replaced two chlorine atoms with cyanuric chloride and two methoxy, two piperidino or two morpholino groups, while the third chlorine was replaced with free α-amino acid. Accordingly, 4,6-dimethoxy-, 4,6-dipiperidino- and 4,6-dimorpholino-1,3,5-triazine-based amino acid derivatives were prepared by subsequent displacement of chlorine atoms.

**Scheme 1:**
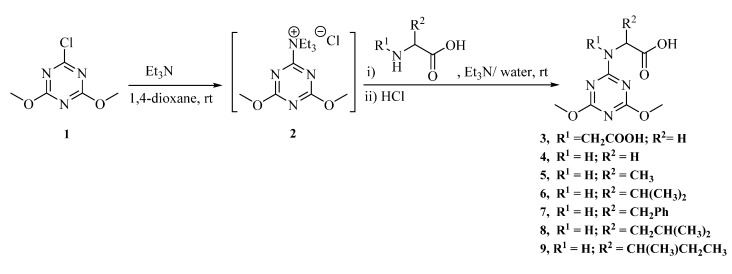
Synthesis of (4,6-dimethoxy-1,3,5-triazin-2-yl) amino acid derivatives **3**–**9**.

The small library of (4,6-dimethoxy-1,3,5-triazin-2-yl) amino acid derivatives **3**–**9** were prepared by reaction of 2-chloro-4,6-dimethoxy triazine **1** and α-amino acids in the presence of triethyl amine as acid scavenger in a 1,4-dioxane/water (1:1) solvent mixture at room temperature (Scheme 1). The reaction was started by addition of *N*,*N*,*N*-triethyl amine (Et_3_N) to a solution of 2-chloro-4,6-dimethoxy triazine **1** in 1,4-dioxane and stirring until a white suspension of 4,6-dimethoxy-1,3,5-triazin-2-yl triethyl ammonium chloride salt **2** was formed. An aqueous solution of α-amino acid and Et_3_N was then added to this white suspension and stirred to give the desired products after neutralization with 5% citric acid or 1 N HCl. The structures of compounds **3**–**9** were confirmed by spectroscopic methods (IR, ^1^H- and ^13^C-NMR) and by elemental analysis.

**Figure 2:**
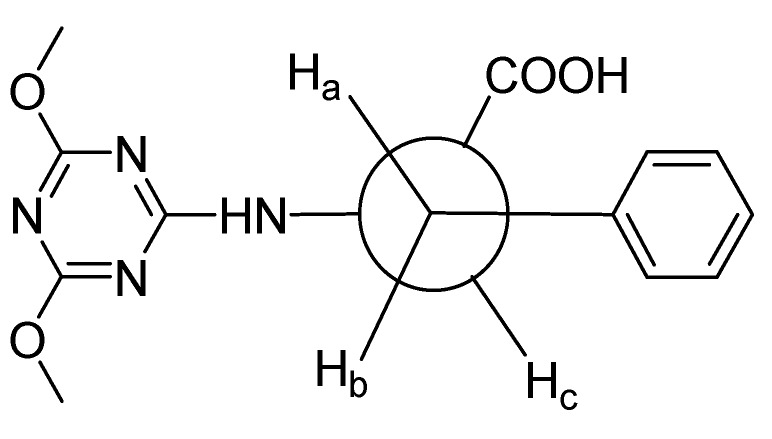
Newman projection formula for 2-(4,6-dimethoxy-1,3,5-triazin-2-ylamino)-3-phenylpropanoic acid **7**.

The ^1^H-NMR spectrum of **7** in DMSO-*d*_6_ (Supplementary Data Figure S9) showed two doublet of doublet (dd) peaks at δ 3.00 ppm and 3.14 ppm, corresponding to the two diastereotopic methylene protons H_a_ and H_b_, respectively, as shown in the staggered conformation using Newman projection (Figure 2).

H_a_ showed a doublet of doublet peak caused by coupling with the germinal proton H_b_ with ^2^*J* = 13.9 Hz, and then with the vicinal proton H_c_ with ^3^*J* = 10.2 Hz (Anti-interaction, dihedral angle = 180°). Similarly, H_b_ showed a doublet of doublet peak as a result of coupling with the germinal proton H_a_ with ^2^*J* = 13.9 Hz, and then with the vicinal proton H_c_ with ^3^*J* = 3.7 Hz (Gauche interaction, dihedral angle = 60°). Two singlet peaks at δ 3.77 ppm and 3.80 ppm corresponding to the two methoxy groups were observed. A multiplet peak appeared at δ 4.54–4.59 ppm corresponding to the α-proton. The peaks corresponding to the aromatic protons appeared as a multiplet at δ 7.18–7.31 ppm. A doublet peak at δ 8.20 ppm with *J* = 8.0 Hz, which is D_2_O-exchangeable, was also observed, corresponding to the NH proton (Supplementary Data Figure S9).

**Scheme 2:**
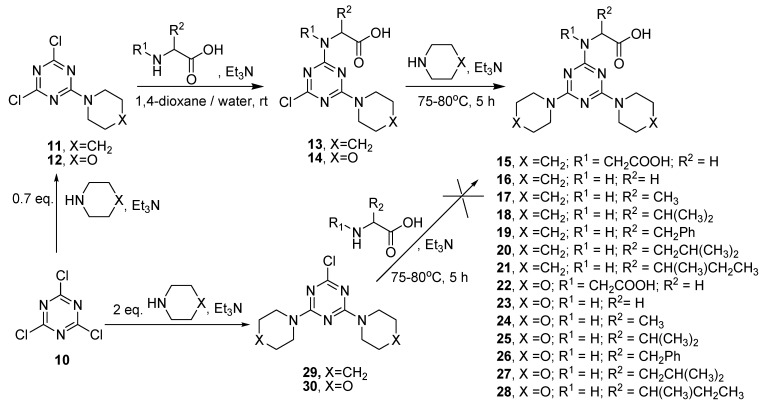
Synthesis of *N*-(4,6-dipiperidino/dimorpholino-1,3,5-triazin-2-yl) amino acid derivatives **15**–**28**.

In addition, *N*-(4,6-dipiperidino/dimorpholino-1,3,5-triazin-2-yl) amino acid derivatives **15**–**28** were prepared through the following sequential reaction: cyanuric chloride **10** was reacted first with piperidine/morpholine in the presence of sodium carbonate (acid scavenger) to afford the corresponding products 2,4-dichloro-6-(piperidin-1-yl)-1,3,5-triazine **11** and 2,4-dichloro-6-morpholino-1,3,5-triazine **12** [28], respectively. Compounds **11** or **12** were allowed to react with free α-amino acids at room temperature. The formed products **13** and **14**, respectively, were then allowed to react directly without isolation with piperidine/morpholine in the presence of Et_3_N, to give the corresponding products, *N*-(4,6-dipiperidino-1,3,5-triazin-2-yl) amino acid derivatives **15**–**21** and *N*-(4,6-dimorpholino-1,3,5-triazin-2-yl) amino acid derivatives **22**–**28** (Scheme 2).

Compounds **29** or **30** were prepared by reaction of cyanuric chloride with equiv. of piperidine/morpholine in the presence of Et_3_N (Scheme 2) [36]. In contrast, the preparation of *N*-(4,6-dipiperidino/dimorpholino-1,3,5-triazin-2-yl) amino acid derivatives through the reaction of free α-amino acids with **29**/**30** was not successful (Scheme 2). The difficulty to displace the third chlorine by the rather weak nucleophilicity of the amino group of α-amino acids can be attributed to the presence of two electron-donating piperidine/morpholine groups, which decreases the positivity of the third chlorine-bearing carbon and prevents the departure of the chlorine atom. The structures of compounds **15**–**28** were confirmed by spectroscopic methods (IR, ^1^H- and ^13^C-NMR) and by elemental analysis (Supplementary Data Figures S15–S35).

### 2.2. Preliminary Biology

The newly synthesized compounds **3**–**9** and **15**–**28** were tested to determine selectivity for MAO-A and MAO-B in the presence of the specific substrate serotonin or benzylamine, respectively. Compounds **7**, **18**, and **25** showed MAO-A inhibition activity comparable to that of the standard clorgyline with apparently more selective inhibitory activity toward MAO-A than MAO-B, and without no significant acute toxicity. More formal studies to confirm these preliminary results will be carried out and published elsewhere.

## 3. Experimental Section 

### 3.1. Chemistry

Solvents and reagents were purchased from Sigma-Aldrich (St. Louis, MO, USA). Unless otherwise stated, the normal workup from organic solvent involved drying over Na_2_SO_4_ and rotary evaporation. TLC was performed using aluminum-backed Merck Silica Gel 60 F-254 plates and suitable solvent systems. Spots were visualized by a Spectroline UV Lamp (254 or 365 nm) or I2 vapor. Melting points were obtained in open capillary tubes using a MEL-Temp II melting point apparatus and uncorrected. Infrared spectra (IR) were recorded on a Perkin-Elmer (Waltham, MA, USA) 1600 series Fourier transform instrument as KBr pellets. The absorption bands (ν_max_) are given in wave numbers (cm^−1^). Nuclear magnetic resonance (NMR) spectra (^1^H-NMR and ^13^C-NMR) were recorded on a JEOL (Tokyo, Japan) 400 MHz and JEOL 500 MHz spectrometer at room temperature. Chemical shifts are reported in parts per million (ppm) and are referenced relative to residual solvent (e.g., CHCl_3_ at δ_H_ 7.26 ppm for CDCl_3_, DMSO at δ_H_ 2.50 ppm for DMSO-*d*_6_). Spin multiplicities are represented by the following signals: singlet (s), broad singlet (br s), doublet (d), broad doublet (br d), doublet of doublets (dd), triplet (t), doublet of triplets (dt), quartet (q), sextet (sex) and multiplet (m). Elemental analyses were performed on a Perkin-Elmer 2400 elemental analyzer, with the values found being within ±0.3% of the theoretical values.

#### 3.1.1. General Procedure for the Synthesis of (4,6-Dimethoxy-1,3,5-triazin-2-yl) Amino Acid Derivatives **3**–**9**

A solution of 2-chloro-4,6-dimethoxy triazine 1 (0.88 g, 5 mmol) and triethyl amine (1.04 mL, 7.5 mmol) in dioxane was stirred at room temperature until a white suspension of 4,6-dimethoxy-1,3,5-trazin-2-yl triethyl ammonium chloride was formed. A solution of α-amino acid (5 mmol) and triethyl amine (1.04 mL, 7.5 mmol) in 6 mL dioxane:water (1:1) was added to the white suspension, forming a clear mixture. The mixture was stirred overnight and then neutralized with 1N HCl to yield a white solid, which was then filtered and dried.

*N-(4,6-Dimethoxy-1,3,5-triazin-2-yl)iminodiacetic acid* (**3**). The product was obtained as a white powder, 6.81 g (83.4%) yield, mp 180–181 °C; IR (KBr): 3500–2589 (br, OH, acid), 1709 (CO, acid) cm^−1^; ^1^H-NMR (400 MHz, DMSO-*d*_6_): δ 3.83 (s, 6H, 2 × OCH_3_), 4.29 (s, 4H, 2 × α-CH_2_), 12.77 (br s, 2H, 2 × COOH); ^13^C-NMR (100 MHz, DMSO-*d*_6_): 50.20, 54.87, 167.77, 171.20, 172.22. Elemental Analysis Calcd. for C_9_H_12_N_4_O_6_: C, 39.71; H, 4.44; N, 20.58. Found: C, 39.76; H, 4.38; N, 20.51.

*2-(4,6-Dimethoxy-1,3,5-triazin-2-ylamino)acetic acid* (**4**). The product was obtained as a white solid, 0.64 g (60.0%) yield; mp: 164–166 °C; IR (KBr): 3574–2522 (br, OH, acid), 3255 (NH, amine), 1725 (CO, acid) cm^−1^; ^1^H-NMR (400 MHz, DMSO-*d*_6_): δ 3.83 (s, 3H, O-CH_3_), 3.80 (s, 3H, O–CH_3_), 3.91 (d, 2H, *J* = 5.9 Hz, CH_2_), 8.13 (t, 1H, *J* = 5.9 Hz, N–H); ^13^C-NMR (100 MHz, DMSO-*d*_6_): 40.67, 54.63, 54.75, 168.56, 171.8, 172.25, 172.36. Elemental Analysis Calcd. for C_7_H_10_N_4_O_4_: C, 39.25; H, 4.71; N, 26.16. Found: C, 39.49; H, 4.45; N, 25.94.

*2-(4,6-Dimethoxy-1,3,5-triazin-2-ylamino)propanoic acid* (**5**). The product was obtained as a white solid, 0.77 g (66.6%) yield; mp: 98–102 °C; IR (KBr): 3557–2567 (br, OH, acid), 3372 (NH, amine), 1721 (CO, acid) cm^−1^; ^1^H-NMR (400 MHz, DMSO-*d*_6_): δ 1.35 (d, 3H, *J* = 7.3 Hz, CH_3_), 3.80 (s, 3H, O–CH_3_), 3.83 (s, 3H, O–CH_3_), 4.34 (quint, 1H, *J* = 7.3 Hz, α-CH), 8.20 (d, 1H, *J* = 7.3 Hz, N–H), 11.18 (br s, 1H, COOH); ^13^C-NMR (100 MHz, DMSO-*d*_6_): 17.30, 49.86, 54.63, 54.75, 167.86, 172.26, 174.85. Elemental Analysis Calcd. for C_8_H_12_N_4_O_4_: C, 42.10; H, 5.30; N, 24.55. Found: C, 42.17; H, 5.22; N, 24.71.

*2-(4,6-Dimethoxy-1,3,5-triazin-2-ylamino)-3-methylbutanoic acid* (**6**). The product was obtained as a white solid, 0.78 g (61.4%) yield; mp: 146–188 °C; IR (KBr): 3570–2539 (br, OH, acid), 3259 (NH, amine), 1720 (CO, acid) cm^−1^; ^1^H-NMR (400 MHz, DMSO-*d*_6_): δ 0.94 (d, 3H, *J* = 6.6 Hz, CH_3_), 0.95 (d, 3H, *J* = 6.6 Hz, CH_3_), 2.13 (octet, 1H, *J* = 6.6 Hz, CH), 3.81 (s, 3H, O–CH_3_), 3.84 (s, 3H, O–CH_3_), 4.21 (t, 1H, *J* = 6.6 Hz, α-CH), 8.03 (d, 1H, *J* = 7.3 Hz, N–H); ^13^C-NMR (100 MHz, DMSO-*d*_6_): 19.11, 19.73, 29.94, 54.65, 54.76, 60.24, 168.48, 172.28, 173.68. Elemental Analysis Calcd. for C_10_H_16_N_4_O_4_: C, 46.87; H, 6.29; N, 21.86. Found: C, 47.02; H, 6.19; N, 21.79.

*2-(4,6-Dimethoxy-1,3,5-triazin-2-ylamino)-3-phenylpropanoic acid* (**7**). The product was obtained as a white solid, 0.98 g (64.1%) yield; mp: 153–155 °C; IR (KBr): 3431–2650 (br, OH, acid), 3256 (NH, amine), 1714 (CO, acid) cm^−1^; ^1^H-NMR (400 MHz, DMSO-*d*_6_): δ 3.00 (dd, 1H, ^2^*J* = 13.9 Hz, ^3^*J* = 10.2 Hz, CH_2_–Ph), 3.14 (dd, 1H, ^2^*J* = 13.9 Hz, ^3^*J* = 3.7 Hz, CH_2_–Ph), 3.77 (s, 3H, OCH_3_), 3.80 (s, 3H, OCH_3_), 4.54–4.59 (m, 1H, α-CH), 7.18–7.31 (m, 5H, Ar–H), 8.20 (d, 1H, *J* = 8.0 Hz, N–H); ^13^C-NMR (100 MHz, DMSO-*d*_6_): 36.74, 54.66, 54.76, 56.11, 126.96, 128.77, 129.62, 138.55, 168.25, 172.21, 173.75. Elemental Analysis Calcd. for C_14_H_16_N_4_O_4_: C, 55.26; H, 5.30; N, 18.41. Found: C, 55.02; H, 5.51; N, 18.55.

*2-(4,6-Dimethoxy-1,3,5-triazin-2-ylamino)-4-methylpentanoic acid* (**8**). The product was obtained as a white solid, 0.91 g (67.4%) yield; mp: 102–103 °C; IR (KBr): 3443–2551 (br, OH, acid), 3282 (NH, amine) 1725 (CO, acid) cm^−1^;^1^H-NMR (400 MHz, DMSO-*d*_6_): δ 0.86 (d, 3H, *J* = 5.9 Hz, CH_3_), 0.90 (d, 3H, *J* = 6.6 Hz, CH_3_), 1.52 (m, 1H, CH), 1.68 (m, 2H, CH_2_), 3.80 (s, 3H, OCH_3_), 3.84 (s, 3H, OCH_3_), 4.35–4.38 (m, 1H, α-CH), 8.16 (d, 1H, *J* = 7.3 Hz, N–H); ^13^C-NMR (100 MHz, DMSO-*d*_6_): 21.67, 23.50, 24.97, 46.18, 52.6, 54.64, 54.75, 168.27, 172.25, 172.31, 174.76. Elemental Analysis Calcd. for C_11_H_18_N_4_O_4_: C, 48.88; H, 6.71; N, 20.73. Found: C, 49.08; H, 6.46; N, 20.97.

*2-(4,6-Dimethoxy-1,3,5-triazin-2-ylamino)-3-methylpentanoic acid* (**9**). The product was obtained as a white solid, 0.98 g (72.5%) yield; mp: 118–119 °C; IR (KBr): 3500–2536 (br, OH, acid), 3261 (NH, amine), 1715 (CO, acid) cm^−1^; ^1^H-NMR (400 MHz, DMSO-*d*_6_): δ 0.85 (t, 3H, *J* = 7.4 Hz, CH_3_CH_2_), 0.91 (d, 3H, *J* = 6.6 Hz, CH_3_CH), 1.26–1.31 (m, 1H, CH_2_), 1.44–1.48 (m, 1H, CH_2_), 1.85–1.88 (m, 1H, CH), 3.81 (s, 3H, OCH_3_), 3.84 (s, 3H, OCH_3_), 4.27 (t, 1H, *J* = 7.4 Hz, α-CH), 8.04 (d, 1H, *J* = 7.4 Hz, N*–*H); ^13^C-NMR (100 MHz, DMSO-*d*_6_): 11.75, 16.14, 25.60, 36.31, 54.65, 54.75, 58.98, 168.35, 172.28, 173.63. Elemental Analysis Calcd. for C_11_H_18_N_4_O_4_: C, 48.88; H, 6.71; N, 20.73. Found: C, 48.69; H, 6.79; N, 20.83.

#### 3.1.2. General Procedure for the Synthesis of *N*-(4,6-Dipiperidino-1,3,5-triazin-2-yl) Amino Acid Derivatives **15**–**21**

A mixture of 2,4-dichloro-6-(piperidin-1-yl)-1,3,5-triazine **11** (0.47 g, 2 mmol) and triethyl amine (0.42 mL, 3 mmol) in 1,4-dioxane (5 mL) was stirred at room temperature until a white suspension was formed. A solution of α-amino acid (2.4 mmol) and triethyl amine (0.42 mL, 3 mmol) in water (2 mL) was added to the suspension to afford a clear mixture. The mixture was stirred overnight at room temperature. Subsequently, piperidine (0.3 mL, 3 mmol) and triethyl amine (0.56 mL, 4 mmol) were added to the reaction mixture and stirred at between 75 °C and 80 °C for 5 h. The reaction mixture was neutralized with 5% citric acid or 1 N HCl. The white precipitate was filtered and recrystallized from ethanol/water to obtain the desired products.

*N-(4,6-Dipiperidino-1,3,5-triazin-2-yl)iminodiacetic acid* (**15**). The product was obtained as a white solid, 0.44 g (79.1%) yield; mp: 128–130 °C; IR (KBr): 3593–2853 (br, OH, acid), 1729 (CO, acid) cm^−1^; ^1^H-NMR (400 MHz, DMSO-*d*_6_): δ 1.44 (br s, 8H, 4 × a-CH_2_), 1.58 (m, 4H, 2 × b-CH_2_), 3.62 (br s, 8H, 4 × CH_2_N), 4.16 (s, 4H, 2 × α-CH_2_), 12.55 (br s, 2H, COOH); ^13^C-NMR (100 MHz, DMSO-*d*_6_): 24.97, 25.90, 40.06, 50.26, 164.81, 165.00, 172.48. Elemental Analysis Calcd. for C_17_H_26_N_6_O_4_: C, 53.96; H, 6.93; N, 22.21. Found: C, 53.77; H, 7.14; N, 22.43.

*2-(4,6-Di(piperidin-1-yl)-1,3,5-triazin-2-ylamino)acetic acid* (**16**). The product was obtained as a white solid, 0.51 g (79.0%) yield; mp: 211–214 °C; IR (KBr): 3628–2664 (br, OH, acid), 3269 (NH, amine) 1676 (CO, acid) cm^−1^; ^1^H-NMR (400 MHz, DMSO-*d*_6_): δ 1.44 (br s, 8H, 4 × a-CH_2_), 1.58 (br s, 4H, 2 × b-CH_2_), 3.62 (br s, 8H, 4 × CH_2_N), 3.79 (d, 2H, *J* = 5.8 Hz, α-CH_2_), 6.85 (t, 1H, *J* = 5.8 Hz, NH), 12.37 (br s, 1H, COOH); ^13^C-NMR (100 MHz, DMSO-*d*_6_): 25.01, 25.93, 42.89, 43.95, 165.04, 166.45, 172.89. Elemental Analysis Calcd. for C_15_H_24_N_6_O_2_: C, 56.23; H, 7.55; N, 26.23. Found: C, 56.03; H, 7.73; N, 26.33.

*2-(4,6-Di(piperidin-1-yl)-1,3,5-triazin-2-ylamino)propanoic acid* (**17**). The product was obtained as a white solid, 0.52 g (78.1%) yield; mp: 126–128 °C; IR (KBr): 3571–2856 (br, OH, acid), 3454 (NH, amine), 1667 (CO, acid) cm^−1^; ^1^H-NMR (400 MHz, DMSO-*d*_6_): δ 1.29 (d, 3H, *J* = 7.3 Hz, CH_3_), 1.43 (br s, 8H, 4 × a-CH_2_), 1.58 (br s, 4H, 2 × b-CH_2_), 3.62 (br s, 8H, 4 × CH_2_N), 4.22 (quint, 1H, *J* = 7.3 Hz, α-CH), 6.82 (d, 1H, *J* = 6.6 Hz, NH); ^13^C-NMR (100 MHz, DMSO-*d*_6_): 19.11, 25.01, 25.94, 43.96, 56.58, 164.00, 165.88, 175.97. Elemental Analysis Calcd. for C_16_H_26_N_6_O_2_: C, 57.46; H, 7.84; N, 25.13. Found: C, 57.59; H, 7.66; N, 25.31.

*2-(4,6-Di(piperidin-1-yl)-1,3,5-triazin-2-ylamino)-3-methylbutanoic acid* (**18**). The product was obtained as a white solid, 0.50 g (70.0%) yield; mp: 183–185 °C; IR (KBr): 3586–2853 (br, OH, acid), 3429 (NH, amine), 1723 (CO, acid) cm^−1^; ^1^H-NMR (500 MHz, DMSO-*d*_6_): δ 0.89 (s, 6H, 2 × CH_3_), 1.39 (br s, 8H, 4 × a-CH_2_), 1.53 (br s, 4H, 2 × b-CH_2_), 2.01 (s, 1H, CH), 3.54 (br s, 8H, 4 × CH_2_N), 4.07 (s, 1H, α-CH), 6.41 (s, 1H, NH, D_2_O exchangeable); Elemental Analysis Calcd. for C_18_H_30_N_6_O_2_: C, 59.64; H, 8.34; N, 23.19. Found: C, 59.59; H, 8.41; N, 23.07.

*2-(4,6-Di(piperidin-1-yl)-1,3,5-triazin-2-ylamino)-3-phenylpropanoic acid* (**19**). The product was obtained as a white solid, 0.67 g (82.0%) yield; mp: 132–134 °C; IR (KBr): 3571–2853 (br, OH, acid), 3316 (NH, amine), 1721 (CO, acid) cm^−1^; ^1^H-NMR (400 MHz, DMSO-*d*_6_): δ 1.42 (br s, 8H, 4 × a-CH_2_), 1.57 (br s, 4H, 2 × b-CH_2_), 2.95–3.07 (m, 2H, CH_2_–Ph), 3.60 (br s, 8H, 4 × CH_2_N), 4.32–4.53 (m, 1H, α-CH), 6.77 (d, 1H, *J* = 7.3 Hz, NH), 7.17–7.31 (m, 5H, Ph–H), 12.5 (br s, 1H, COOH); ^13^C-NMR (100 MHz, DMSO-*d*_6_): 24.99, 25.93, 37.00, 43.96, 56.14, 126.80, 128.69, 129.68, 138.99, 164.77, 164.94, 166.14, 174.90. Elemental Analysis Calcd. for C_22_H_30_N_6_O_2_: C, 64.37; H, 7.37; N, 20.47. Found: C, 64.18; H, 7.43; N, 20.67.

*2-(4,6-Di(piperidin-1-yl)-1,3,5-triazin-2-ylamino)-4-methylpentanoic acid* (**20**). The product was obtained as a white solid, 0.54 g (71.8%) yield; mp:158–161 °C; IR (KBr): 3614–2855 (br, OH, acid), 3320 (NH, amine) 1721 (CO, acid) cm^−1^; ^1^H-NMR (400 MHz, DMSO-*d*_6_): δ 0.86 (d, 3H, *J* = 6.6 Hz, CH_3_), 0.88 (d, 3H, *J* = 6.6 Hz, CH_3_), 1.38–1.49 (m, 1H, CH), 1.44 (br s, 8H, 4 × a-CH_2_), 1.57 (br s, 4H, 2 × b-CH_2_), 1.57–1.73 (m, 2H, CH_2_), 3.62–3.64 (m, 8H, 4 × CH_2_N), 4.27–4.29 (m, 1H, α-CH), 6.78 (d, 1H, *J* = 7.3 Hz, NH), 12.26 (br s, 1H, COOH); ^13^C-NMR (100 MHz, DMSO-*d*_6_): 21.87, 23.59, 24.90, 25.02, 25.89, 26.01, 43.94, 52.36, 164.99, 166.30, 175.88. Elemental Analysis Calcd. for C_19_H_32_N_6_O_2_: C, 60.61; H, 8.57; N, 22.32. Found: C, 60.45; H, 8.72; N, 22.16.

*2-(4,6-Di(piperidin-1-yl)-1,3,5-triazin-2-ylamino)-3-methylpentanoic acid* (**21**). The product was obtained as a white solid, 0.60 g (79.7%) yield; mp:176–178 °C; IR (KBr): 3614–2854 (br, OH, acid), 3315 (NH, amine) 1726 (CO, acid) cm^−1^; ^1^H-NMR (400 MHz, DMSO-*d*_6_): δ 0.85 (t, 3H, *J* = 6.6 Hz, CH_3_), 0.88 (d, 3H, *J* = 6.6 Hz, CH_3_), 1.22–1.52 (m, 2H, CH_2_), 1.44 (br s, 8H, 4 × a-CH_2_), 1.58 (br s, 4H, 2 × b-CH_2_), 1.77–1.82 (m, 1H, CH), 3.63 (br s, 8H, 4 × CH_2_N), 4.17 (t, 1H, *J* = 7.3 Hz, α-CH), 6.51 (d, 1H, *J* = 7.3 Hz, NH), 12.33 (br s, 1H, COOH); ^13^C-NMR (100 MHz, DMSO-*d*_6_): 11.73, 16.22, 25.01, 25.91, 26.02, 36.46, 43.95, 58.64, 164.84, 164.99, 166.30, 174.68. Elemental Analysis Calcd. for C_19_H_32_N_6_O_2_: C, 60.61; H, 8.57; N, 22.32. Found: C, 60.83; H, 8.36; N, 22.16.

#### 3.1.3. General Procedure for the Synthesis of *N*-(4,6-Dimorpholino-1,3,5-triazin-2-yl) Amino Acid Derivatives **22**–**28**

The mixture of 2,4-dichloro-6-morpholino-1,3,5-triazine **12** (0.47 g, 2 mmol) and triethyl amine (0.42 mL, 3 mmol) in 1,4-dioxane (5 mL) was stirred at room temperature until a white suspension was formed. A solution of α-amino acid (1.2 equiv.) and triethyl amine (0.42 mL, 3 mmol) in water (2 mL) was added to the suspension to afford a clear mixture. The mixture was stirred overnight at room temperature. Subsequently, morpholine (0.26 mL, 3 mmol) and triethyl amine (0.56 mL, 4 mmol) were added to the reaction mixture and stirred at between 75 °C and 80 °C for 5 h. The reaction mixture was neutralized with 5% citric acid or 1 N HCl. The white precipitate was filtered and recrystallized from ethanol/water to obtain the desired products.

*N-(4,6-Dimorpholino-1,3,5-triazin-2-yl)iminodiacetic acid* (**22**). The product was obtained as a white solid, 0.48 g (63.0%) yield; mp: 260 °C (decom.); IR (KBr): 3700–2660 (br, OH, acid), 1718 (CO, acid) cm^−^^1^; ^1^H-NMR (500 MHz, DMSO-*d*_6_): δ 3.50–3.53 (m, 8H, 4 × CH_2_N), 3.55–3.57 (m, 8H, 4 × CH_2_O), 3.94 (s, 4H, 2 × α-CH_2_), 11.63 (br s, 2H, 2 × COOH). Elemental Analysis Calcd for C_15_H_22_N_6_O_6_: C, 47.12; H, 5.80; N, 21.98. Found: C, 47.02; H, 5.94; N, 22.09.

*2-(4,6-Dimorpholino-1,3,5-triazin-2-ylamino)acetic acid* (**23**)*.* The product was obtained as a white solid, 0.44 g (68.0%) yield; mp: 224–226 °C; IR (KBr): 3436 (br, OH, acid), 3296 (NH, amine), 1679 (CO, acid) cm^−1^; ^1^H-NMR (500 MHz, DMSO-*d*_6_): δ 3.54 (t, 8H, *J* = 4.6 Hz, 4 × CH_2_N), 3.58 (t, 8H, *J* = 4.6 Hz, 4 × CH_2_O), 3.78 (d, 2H, *J* = 6.1 Hz, α-CH_2_), 6.98 (t, 1H, *J* = 6.1 Hz, N–H), 11.92 (br s, 1H, COOH); ^13^C-NMR (125 MHz, DMSO-*d*_6_): 43.66, 44.82, 66.55, 165.05, 165.23, 166.29, 172.76. Elemental Analysis Calcd. for C_13_H_20_N_6_O_4_: C, 48.14; H, 6.22; N, 25.91. Found: C, 48.01; H, 6.34; N, 26.07.

*2-(4,6-Dimorpholino-1,3,5-triazin-2-ylamino)propanoic acid* (**24**). The product was obtained as a white solid, 0.42 g (61.8%) yield; mp: 194–196 °C; IR (KBr): 3642–2860 (br, OH, acid), 3428 (NH, amine), 1681 (CO, acid) cm^−1^; ^1^H-NMR (500 MHz, DMSO-*d*_6_): δ 1.26 (d, 3H, *J* = 7.7 Hz, CH_3_), 3.53–3.56 (m, 8H, 4 × CH_2_N), 3.57–3.3.61 (m, 8H, 4 × CH_2_O), 4.19 (quint, 1H, *J* = 6.9 Hz, α-CH), 6.99 (d, 1H, *J* = 6.9 Hz, NH), 12.07 (br s, 1H, COOH). Elemental Analysis Calcd. for C_14_H_22_N_6_O_4_: C, 49.70; H, 6.55; N, 24.84. Found: C, 49.59; H, 6.77; N, 24.91.

*2-(4,6-Dimorpholino-1,3,5-triazin-2-ylamino)-3-methylbutanoic acid* (**25**). The product was obtained as a white solid, 0.49 g (67.5%) yield; mp: 170–172 °C; IR (KBr): 3438 (br, OH, acid), 3305 (NH, amine), 1723 (CO, acid) cm^−1^; ^1^H-NMR (500 MHz, DMSO-*d*_6_): δ 0.88 (d, 3H, *J* = 7.7 Hz, CH_3_), 0.89 (d, 3H, *J* = 6.9 Hz, CH_3_), 1.97–2.04 (m, 1H, CH), 3.53 (s, 8H, 4 × CH_2_N), 3.60 (s, 8H, 4 × CH_2_O), 4.05 (t, 1H, *J* = 6.9 Hz, α-CH), 6.73 (d, 1H, *J* = 6.9 Hz, NH). Elemental Analysis Calcd. for C_16_H_26_N_6_O_4_: C, 52.45; H, 7.15; N, 22.94. Found: C, 52.29; H, 6.99; N, 23.05.

*2-(4,6-Dimorpholino-1,3,5-triazin-2-ylamino)-3-phenylpropanoic acid* (**26**). The product was obtained as a white solid, 0.69 g (83.1%) yield; mp: 126–128 °C; IR (KBr): 3642–2855 (br, OH, acid), 3427 (NH, amine), 1729 (CO, acid) cm^−1^; ^1^H-NMR (500 MHz, DMSO-*d*_6_) isomer A (83.0%): δ 2.91–2.99 (m, 1H, CH_2_–Ph), 3.01 (dd, 1H, *J* = 13.8 Hz, *J* = 5.4 Hz, CH_2_–Ph), 3.53 (s, 8H, 4 × CH_2_N), 3.57 (t, 8H, *J* = 5.4 Hz, 4 × CH_2_O), 4.37–4.41 (m, 1H, α-CH), 6.92 (d, 1H, *J* = 7.7 Hz, NH), 7.10–7.24 (m, 5H, Ph–H); isomer B (17.0%): δ 2.76–2.90 (m, 2H, CH_2_–Ph), 3.53 (s, 8H, 4 × CH_2_N), 3.57 (t, 8H, *J* = 5.4 Hz, 4 × CH_2_O), 4.52–4.54 (m, 1H, α-CH), 6.82 (d, 1H, *J* = 8.4 Hz, NH), 7.10–7.24 (m, 5H, Ph–H).Elemental Analysis Calcd. for C_20_H_26_N_6_O_4_: C, 57.96; H, 6.32; N, 20.28. Found: C, 57.76; H, 6.51; N, 20.33.

*2-(4,6-Dimorpholino-1,3,5-triazin-2-ylamino)-4-methylpentanoic acid* (**27**). The product was obtained as a white solid, 0.48 g (62.7%) yield; mp: 120–122 °C; IR (KBr): 3720–2500 (br, OH, acid), 3426 (NH, amine), 1728 (CO, acid) cm^−1^; ^1^H-NMR (500 MHz, DMSO-*d*_6_): δ 0.82 (d, 3H, *J* = 6.1 Hz, CH_3_), 0.85 (d, 3H, *J* = 6.1 Hz, CH_3_), 1.38–1.44 (m, 1H, CH), 1.57–1.67 (m, 2H, CH_2_), 3.53–3.55 (m, 8H, 4 × CH_2_N), 3.56–3.60 (m, 8H, 4 × CH_2_O), 4.21–4.25 (m, 1H, α-CH), 6.94 (d, 1H, *J* = 6.9 Hz, NH), 12.13 (br.s, 1H, COOH). Elemental Analysis Calcd. for C_17_H_28_N_6_O_4_: C, 53.67; H, 7.42; N, 22.09. Found: C, 53.54; H, 7.34; N, 22.19.

*2-(4,6-Dimorpholino-1,3,5-triazin-2-ylamino)-3-methylpentanoic acid* (**28**). The product was obtained as a white solid, 0.65 g (85.6%) yield; mp: 96–98 °C; IR (KBr): 3609–2661 (br, OH, acid), 3485 (NH, amine), 1670 (CO, acid) cm^−1^; ^1^H-NMR (500 MHz, CDCl_3_): δ 0.87–0.92 (m, 3H, CH_3_CH_2_), 0.93 (d, 3H, *J* = 6.2 Hz, CH_3_CH), 1.15–1.29 (m, 1H, CH_2_), 1.42–1.61 (m, 1H, CH_2_), 1.96–2.01 (m, 1H, CH), 3.66–3.74 (m, 8H, 4 × CH_2_N), 3.75–3.84 (m, 8H, 4 × CH_2_O), 4.50–4.64 (m, 1H, α-CH), 7.56–7.78 (m, 1H, NH), 8.24 (br s, 1H, COOH). Elemental Analysis Calcd. for C_17_H_28_N_6_O_4_: C, 53.67; H, 7.42; N, 22.09. Found: C, 53.78; H, 7.33; N, 21.98.

## 4. Conclusions

The synthesis and a preliminary biochemical evaluation of the newly synthesized *N*-(2,4-disubstituted-1,3,5-triazin-2-yl) amino acid derivatives as MAO inhibitors were described. Compounds **7**, **18** and **25** showed the highest activity within the test compounds comparable to that of the standard clorgyline. These preliminary tests have also shown remarkable selectivity within the test compounds as MAO-A inhibitors. Therefore, such compounds would represent a fruitful matrix for the development of a new class of MAO-A inhibitors that would deserve further investigation and derivatization.

## Supplementary Materials

Supplementary materials can be accessed at: http://www.mdpi.com/1420-3049/20/09/15976/s1.
