# Supplementary material for: Synthesis and Preliminary Biological Evaluation of 1,3,5-Triazine Amino Acid Derivatives to Study Their MAO Inhibitors
Source: Molecules. 2015 Sep 2;20(9):15976–88. doi: 10.3390/molecules200915976 (PMC6332092; doi:10.3390/molecules200915976)
Supplement: Supplementary file 1 [file molecules-20-15976-s001.pdf]

Supplementary Materials

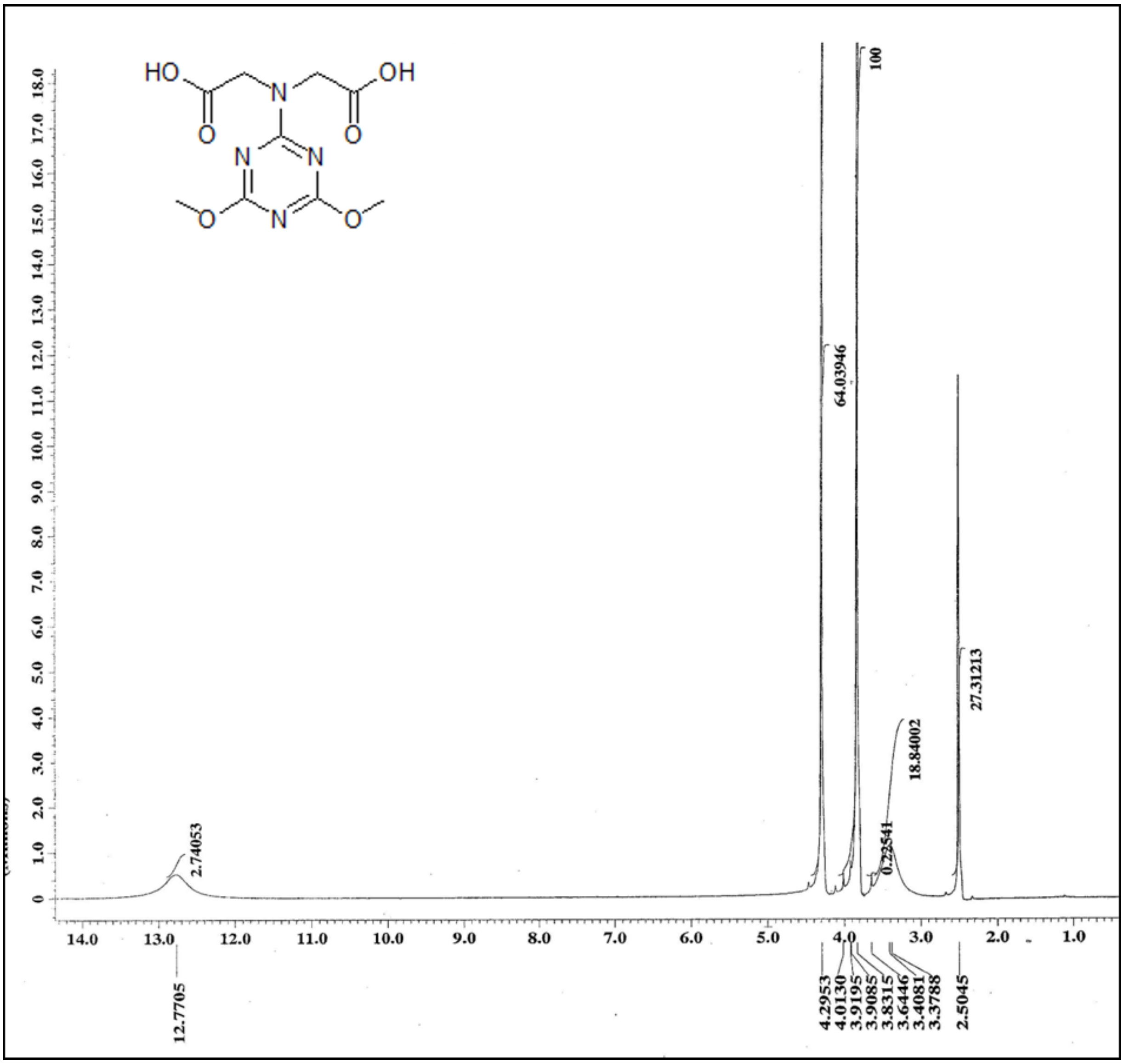

Figure S1. ^{1}H-NMR (DMSO-d_6) spectra of *N*-(4,6-dimethoxy-1,3,5-triazin-2-yl)iminodiacetic acid **4**.

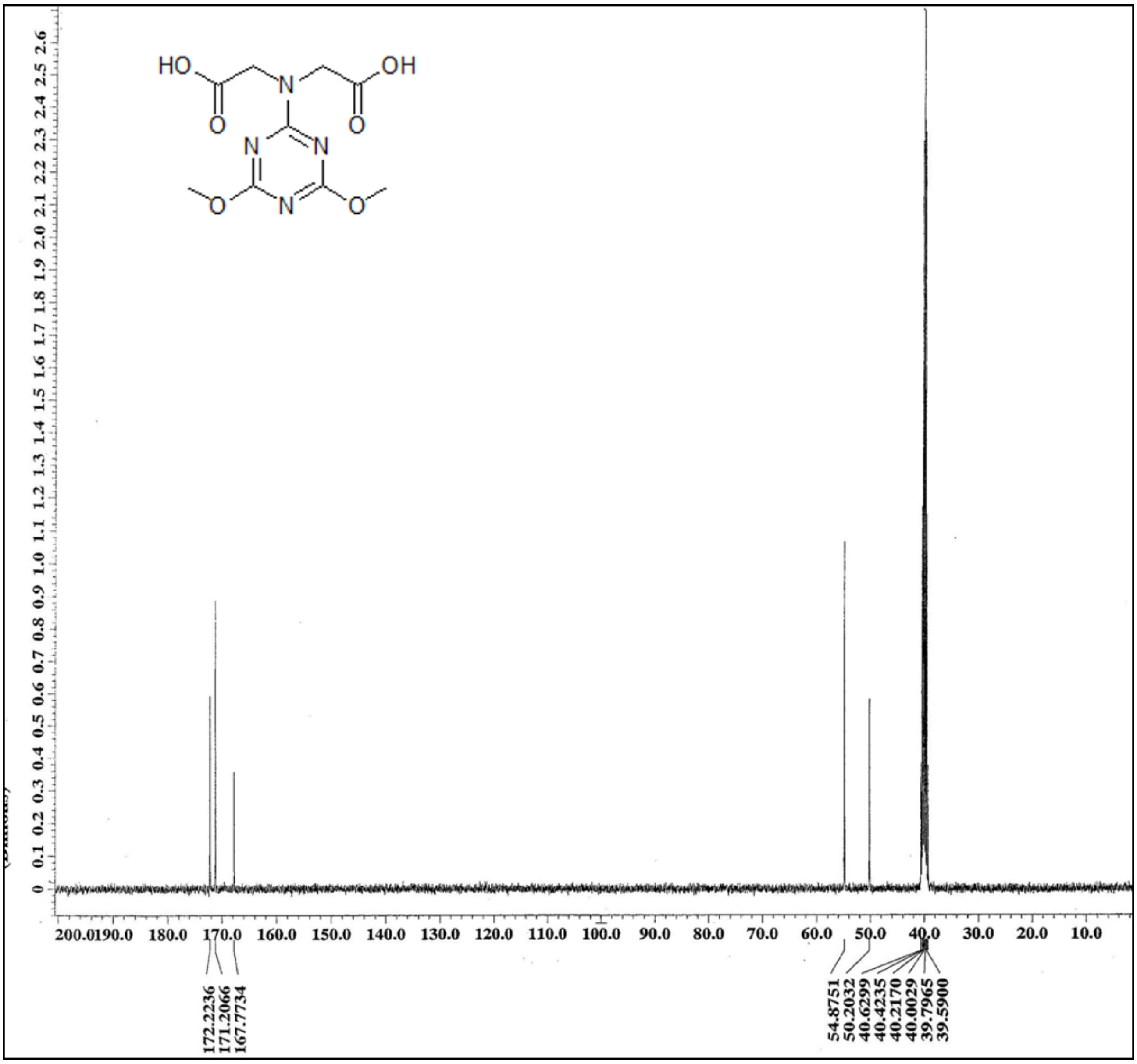

Figure S2. ^{13}C-NMR (DMSO-d_6) spectra of *N*-(4,6-dimethoxy-1,3,5-triazin-2-yl)iminodiacetic acid **4**.

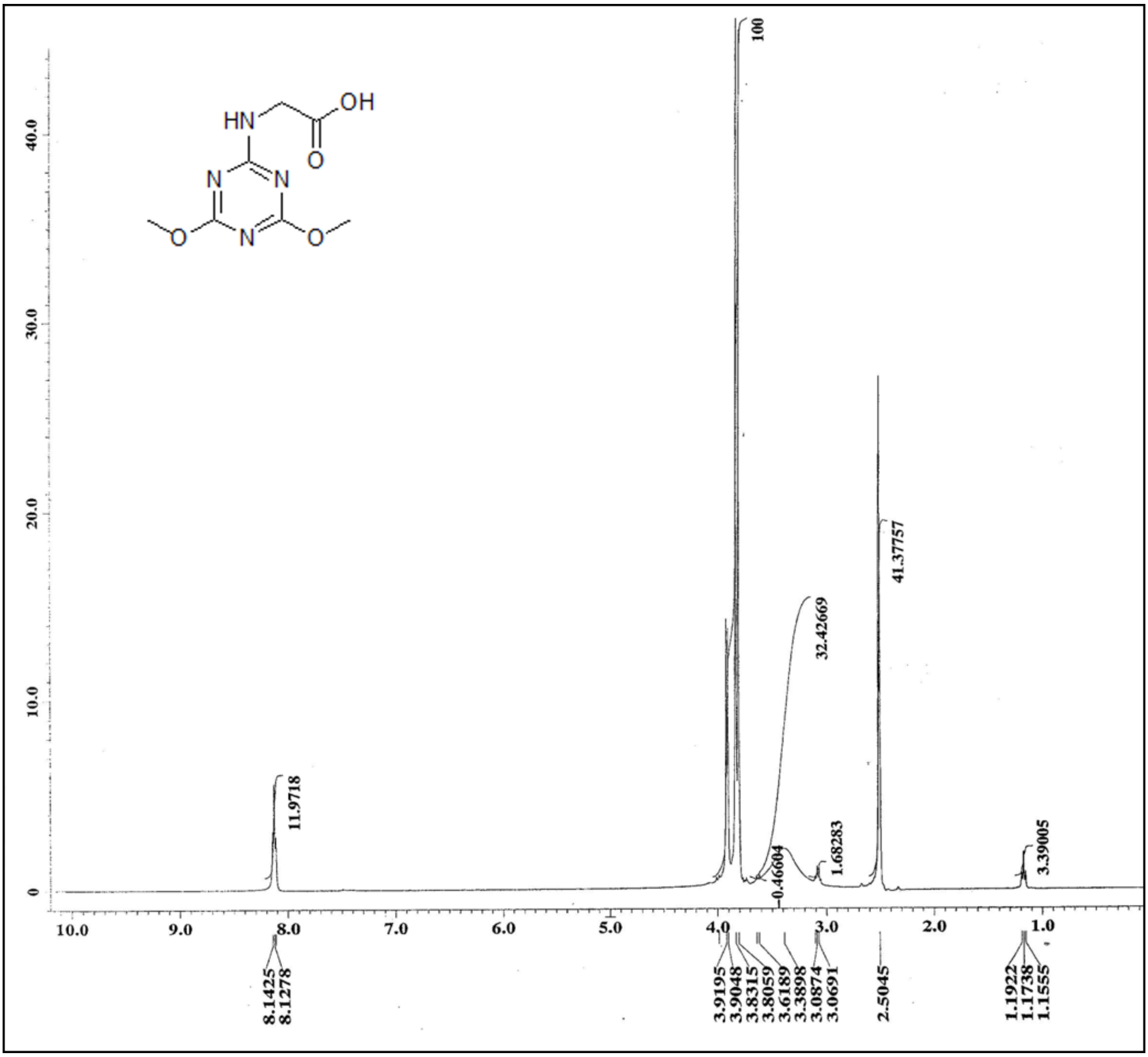

Figure S3. ^{1}H-NMR (DMSO-d_6) spectra of 2-(4,6-dimethoxy-1,3,5-triazin-2-ylamino)acetic acid **5**.

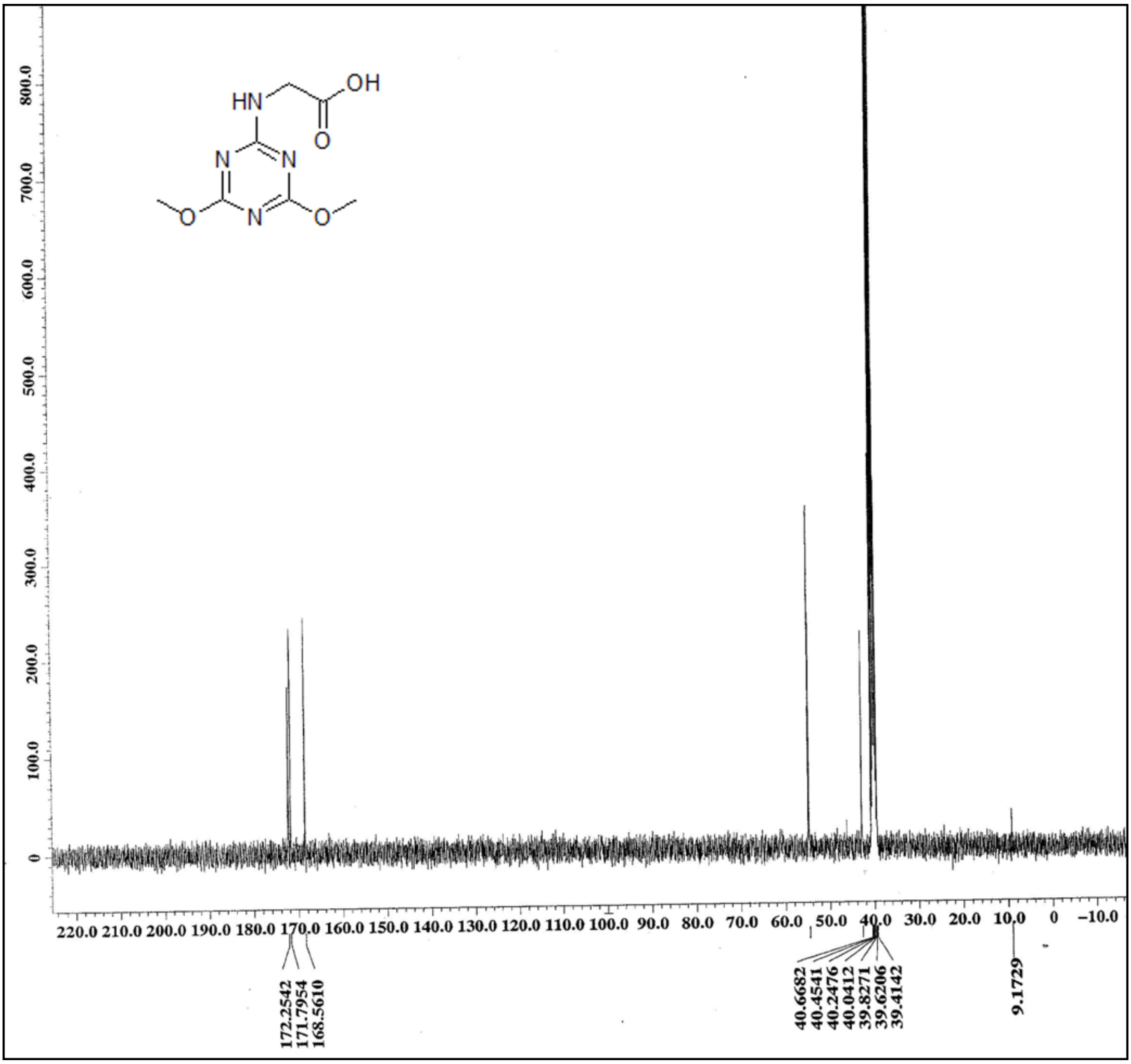

Figure S4. ^{13}C-NMR (DMSO-d_6) spectra of 2-(4,6-dimethoxy-1,3,5-triazin-2-ylamino)acetic acid **5**.

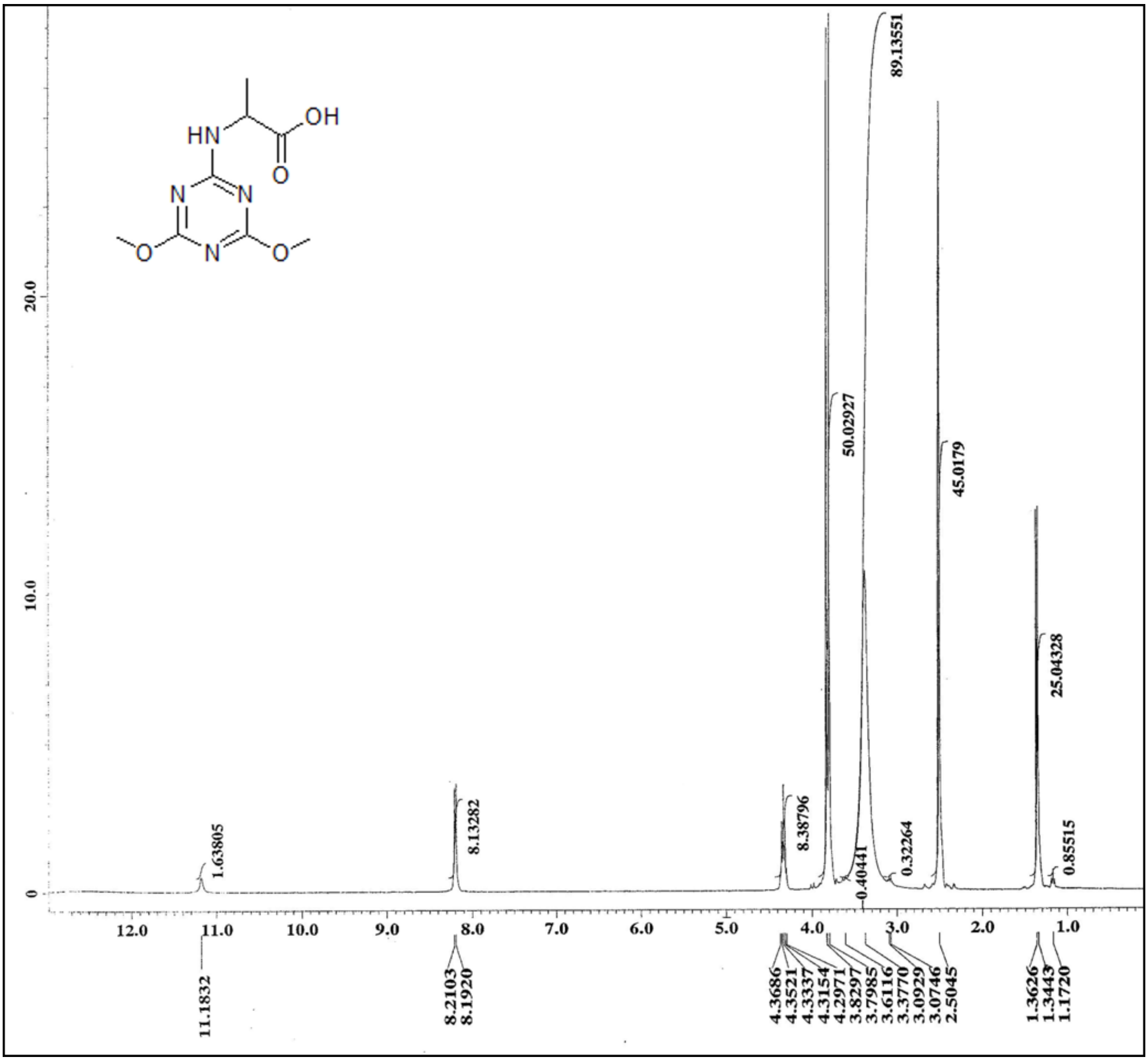

Figure S5. ^{1}H-NMR (DMSO-d_6) spectra of 2-(4,6-dimethoxy-1,3,5-triazin-2-ylamino)propanoic acid **6**.

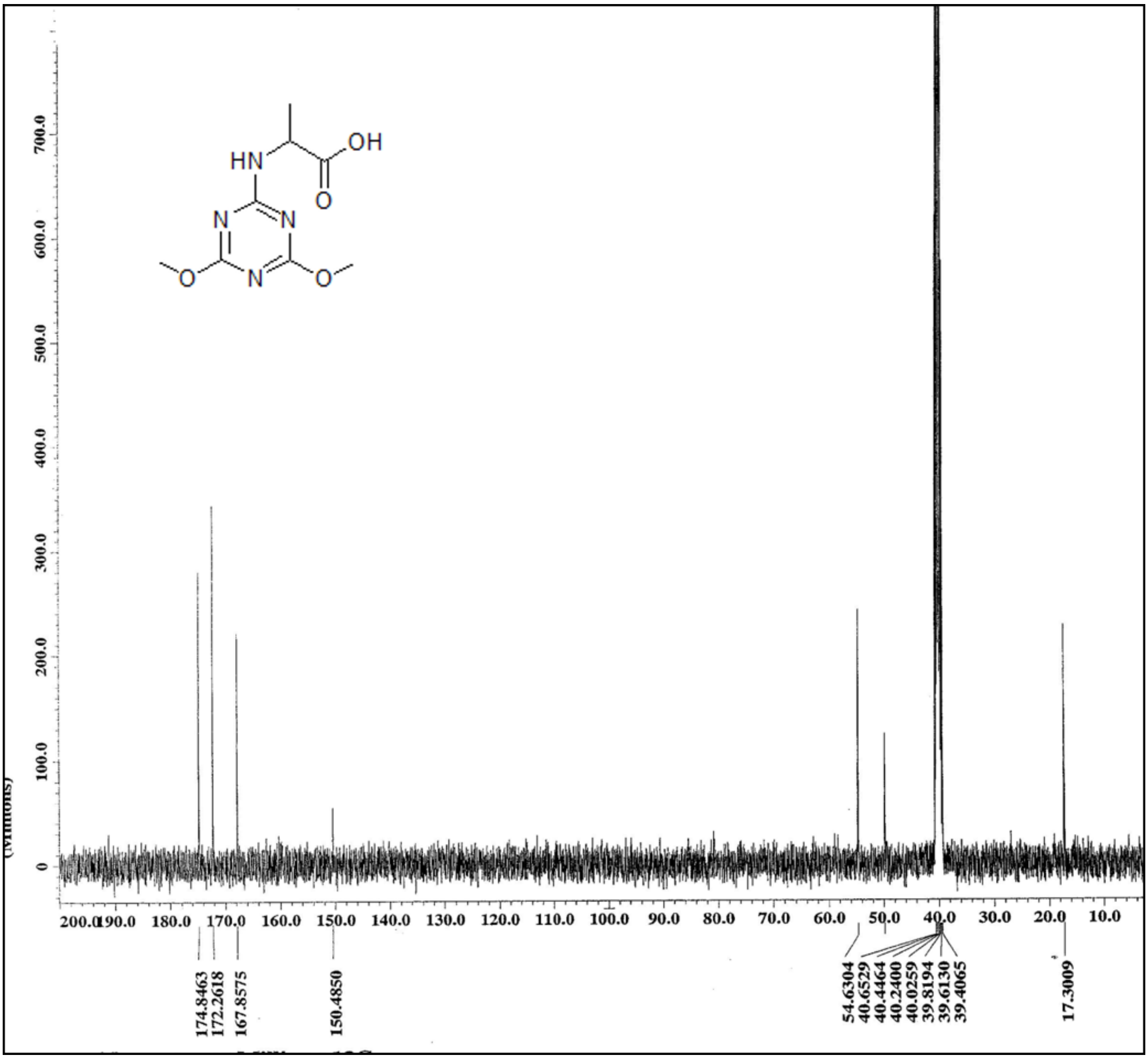

Figure S6. ^{13}C-NMR (DMSO-d_6) spectra of 2-(4,6-dimethoxy-1,3,5-triazin-2-ylamino)propanoic acid **6**.

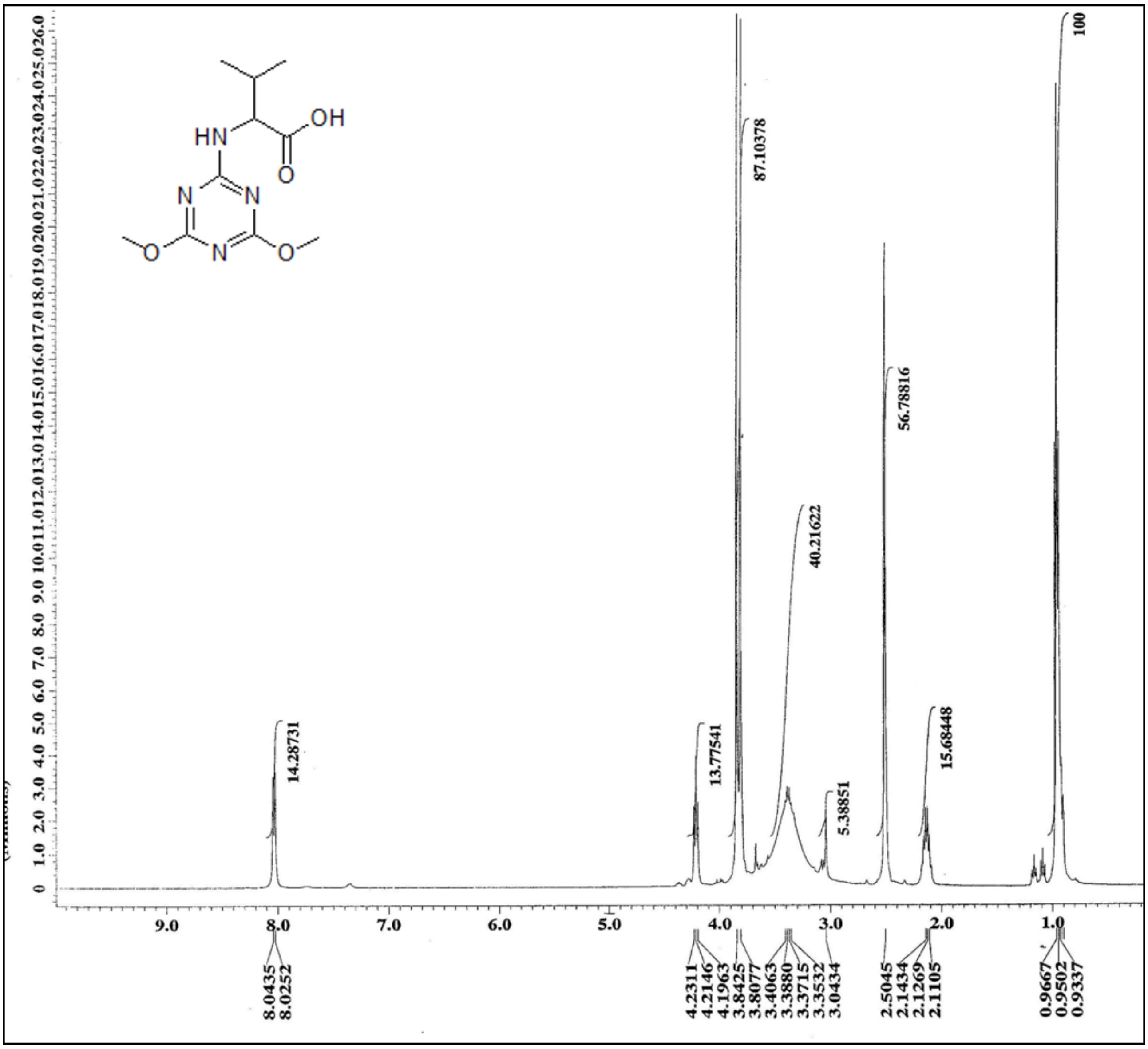

Figure S7. ^{1}H-NMR (DMSO-d_6) spectra of 2-(4,6-dimethoxy-1,3,5-triazin-2-ylamino)-3-methylbutanoic acid **7**.

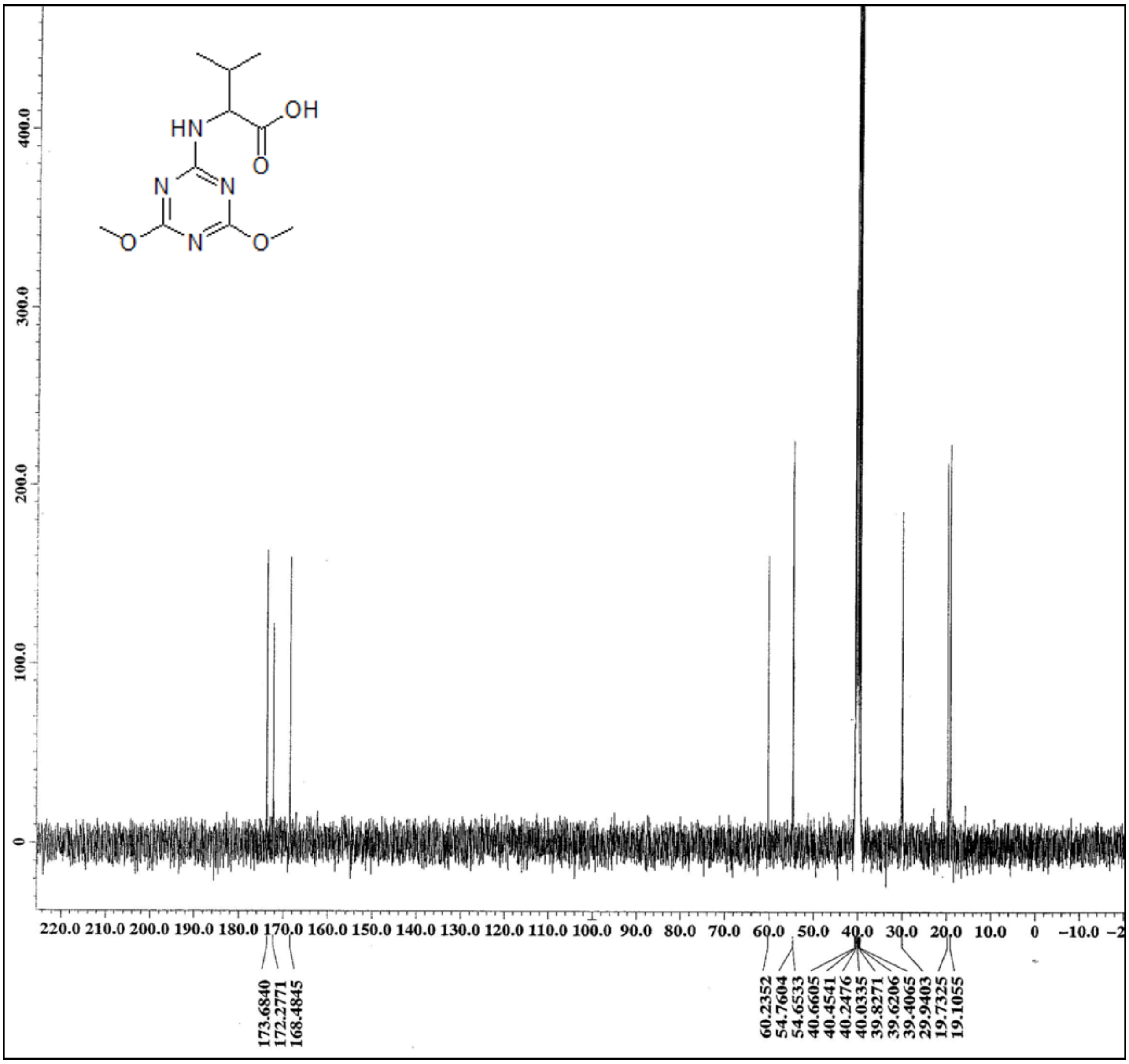

Figure S8. ^{13}C-NMR (DMSO-d_6) spectra of 2-(4,6-dimethoxy-1,3,5-triazin-2-ylamino)-3-methylbutanoic acid **7**.

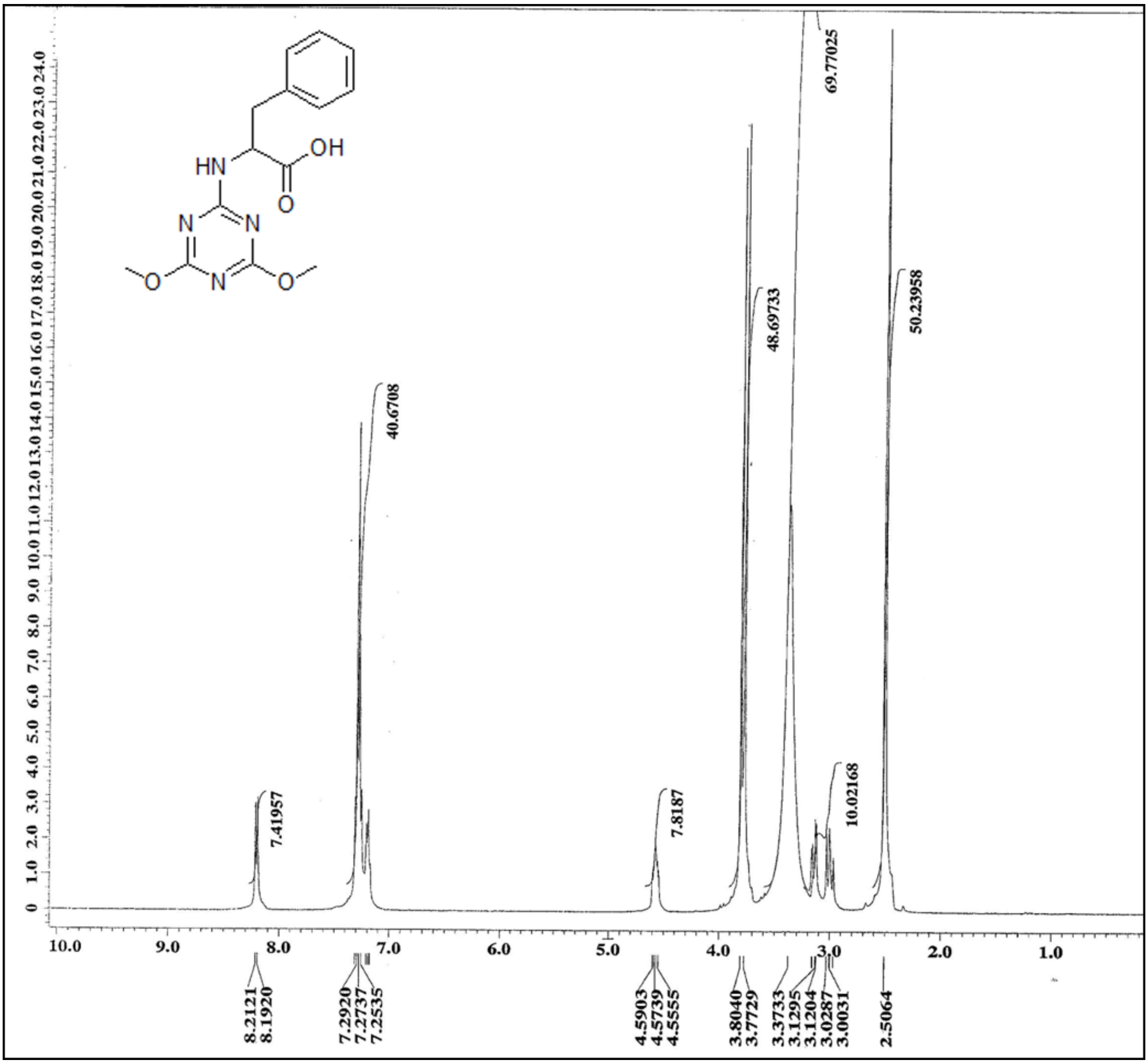

Figure S9. ^{1}H-NMR (DMSO-d_6) spectra of 2-(4,6-dimethoxy-1,3,5-triazin-2-ylamino)-3-phenylpropanoic acid **8**.

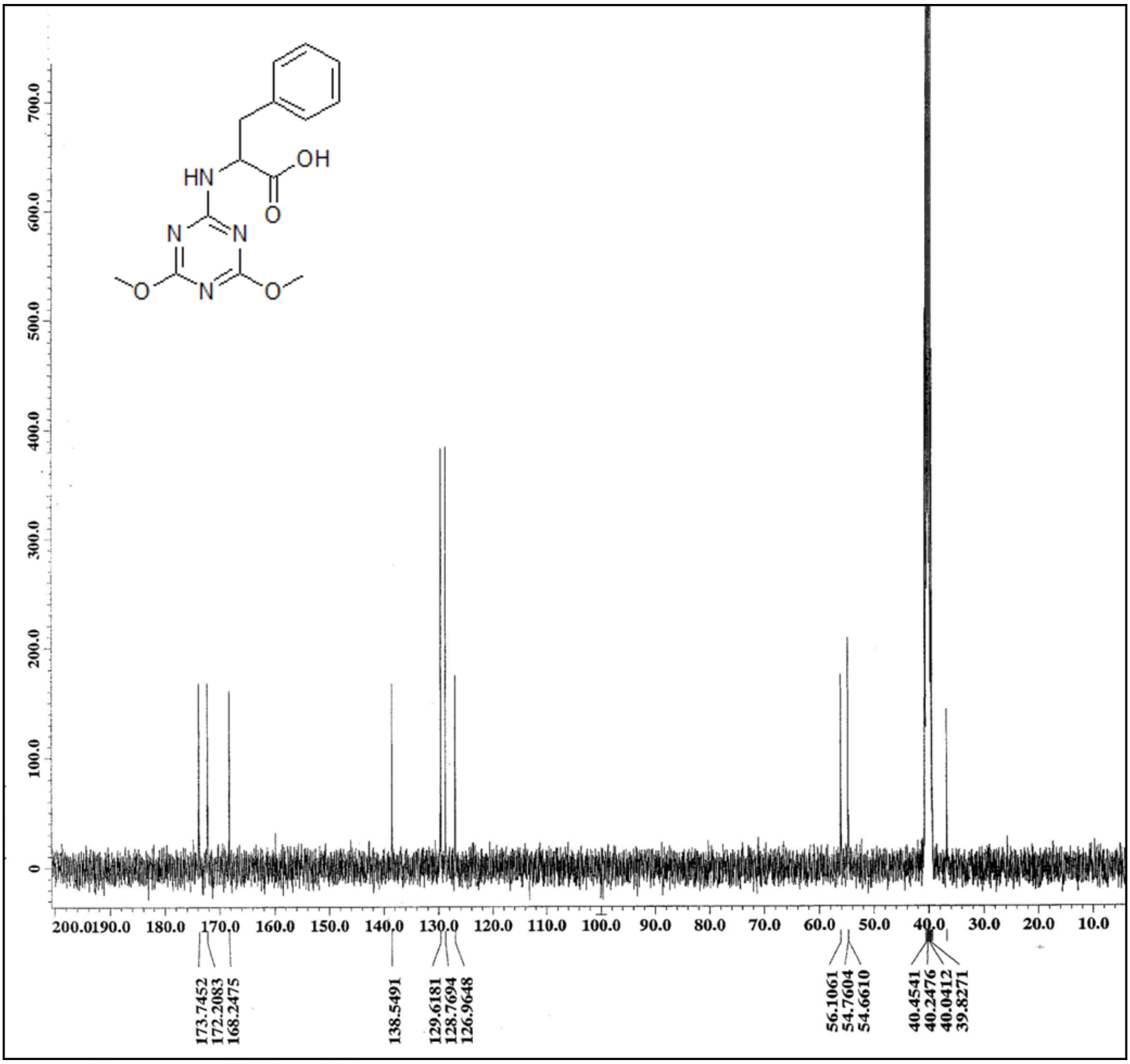

Figure S10. ^{13}C-NMR (DMSO-d_6) spectra of 2-(4,6-dimethoxy-1,3,5-triazin-2-ylamino)-3-phenylpropanoic acid **8**.

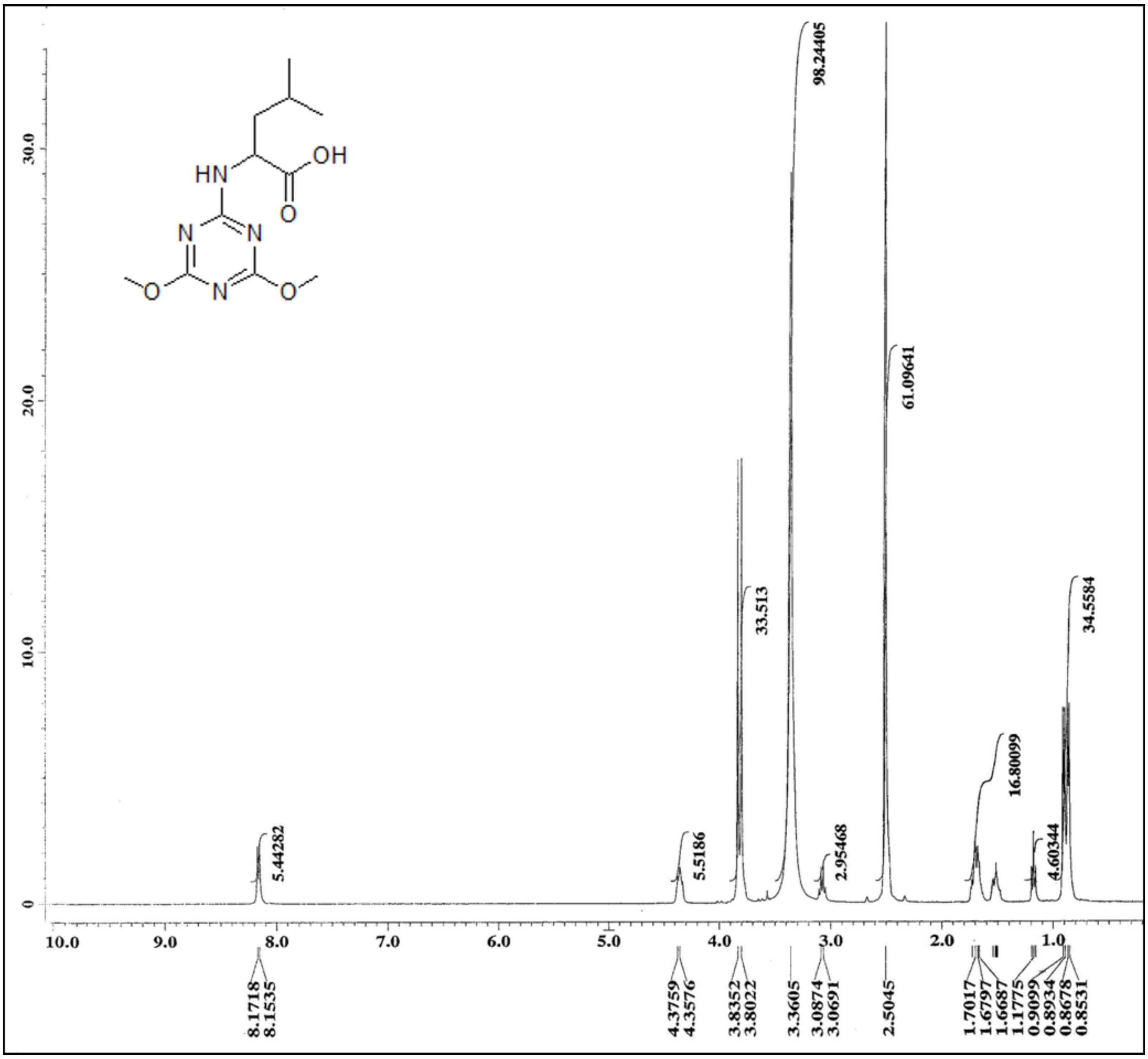

Figure S11. ^{1}H-NMR (400 MHz, DMSO-d_6) spectra of 2-(4,6-dimethoxy-1,3,5-triazin-2-ylamino)-4-methylpentanoic acid **9**.

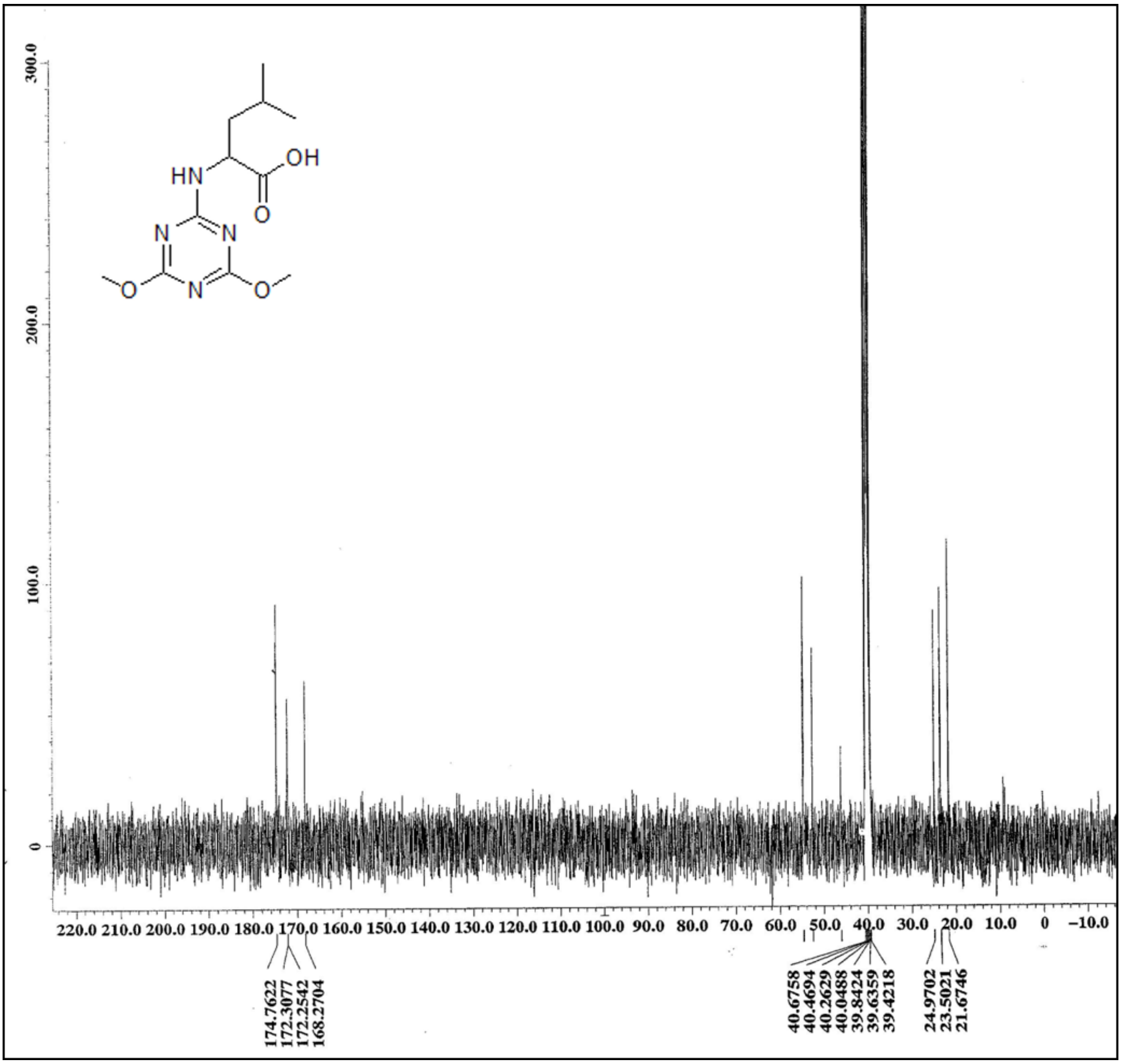

Figure S12. ^{13}C-NMR (400 MHz, DMSO-*d*$_6$) spectra of 2-(4,6-dimethoxy-1,3,5-triazin-2-ylamino)-4-methylpentanoic acid **9**.

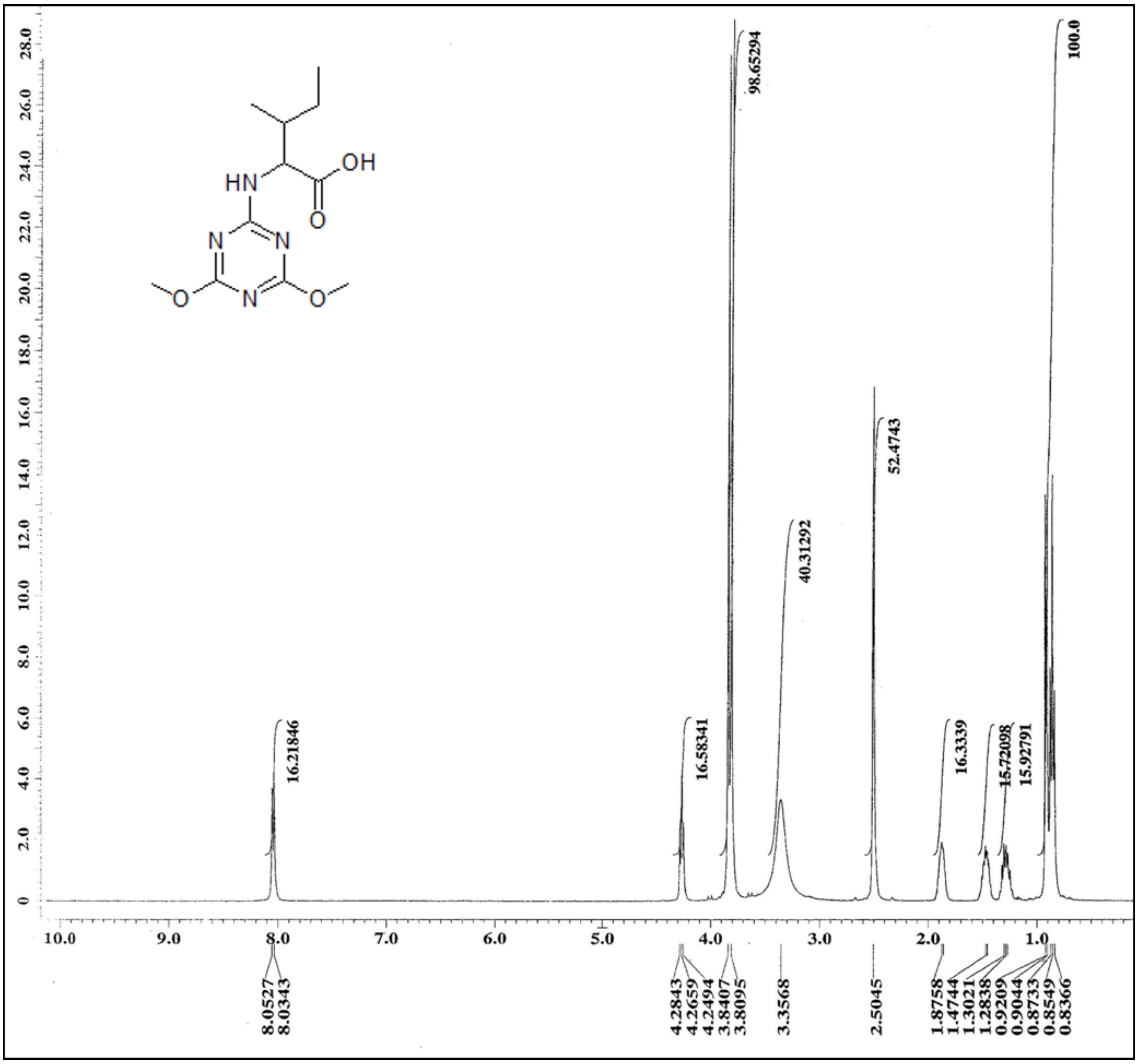

Figure S13. ^{1}H-NMR (400 MHz, DMSO-d_6) spectra of 2-(4,6-dimethoxy-1,3,5-triazin-2-ylamino)-3-methylpentanoic acid **10**.

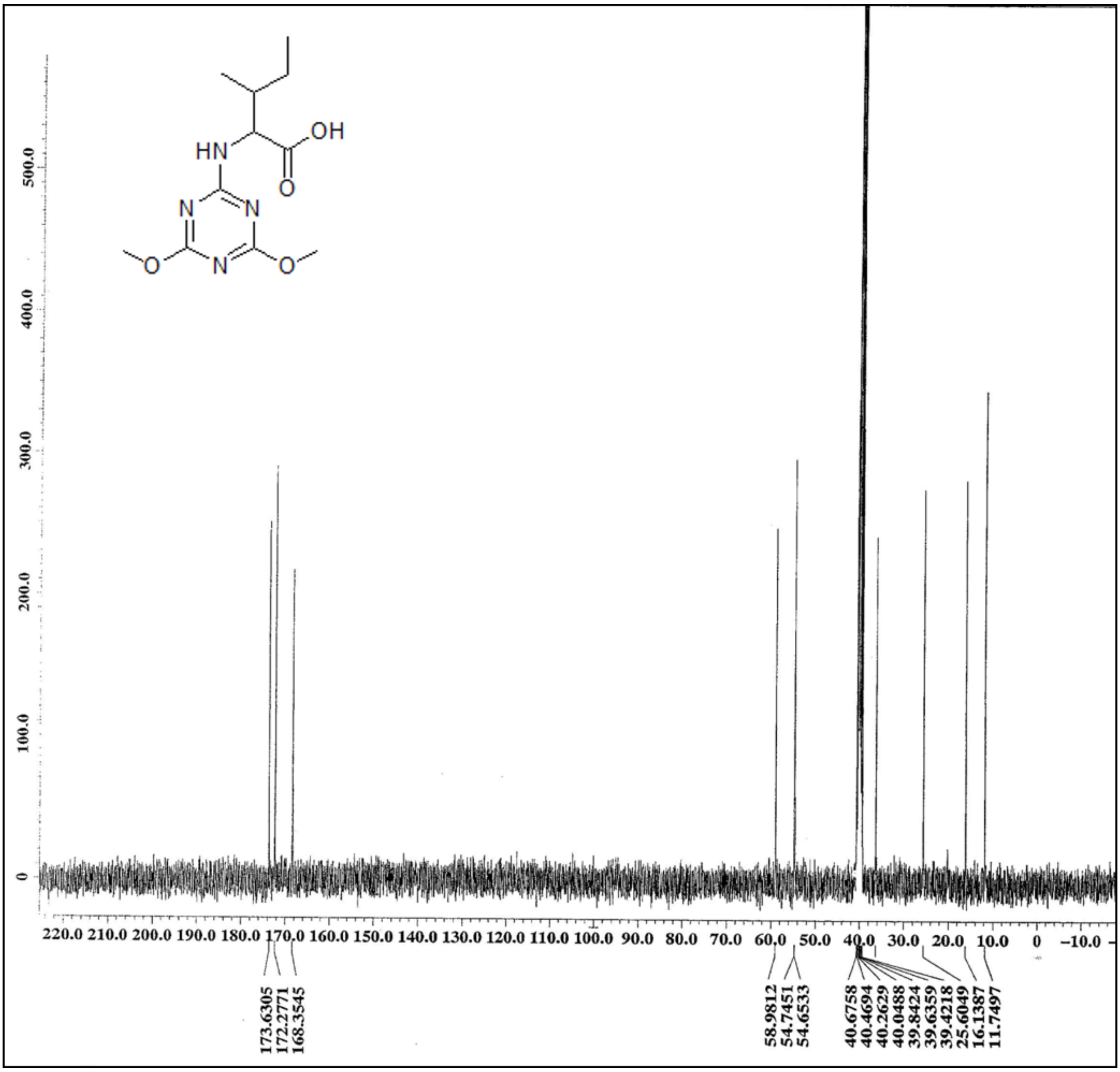

Figure S14. ^{13}C-NMR (400 MHz, DMSO-d_6) spectra of 2-(4,6-dimethoxy-1,3,5-triazin-2-ylamino)-3-methylpentanoic acid **10**.

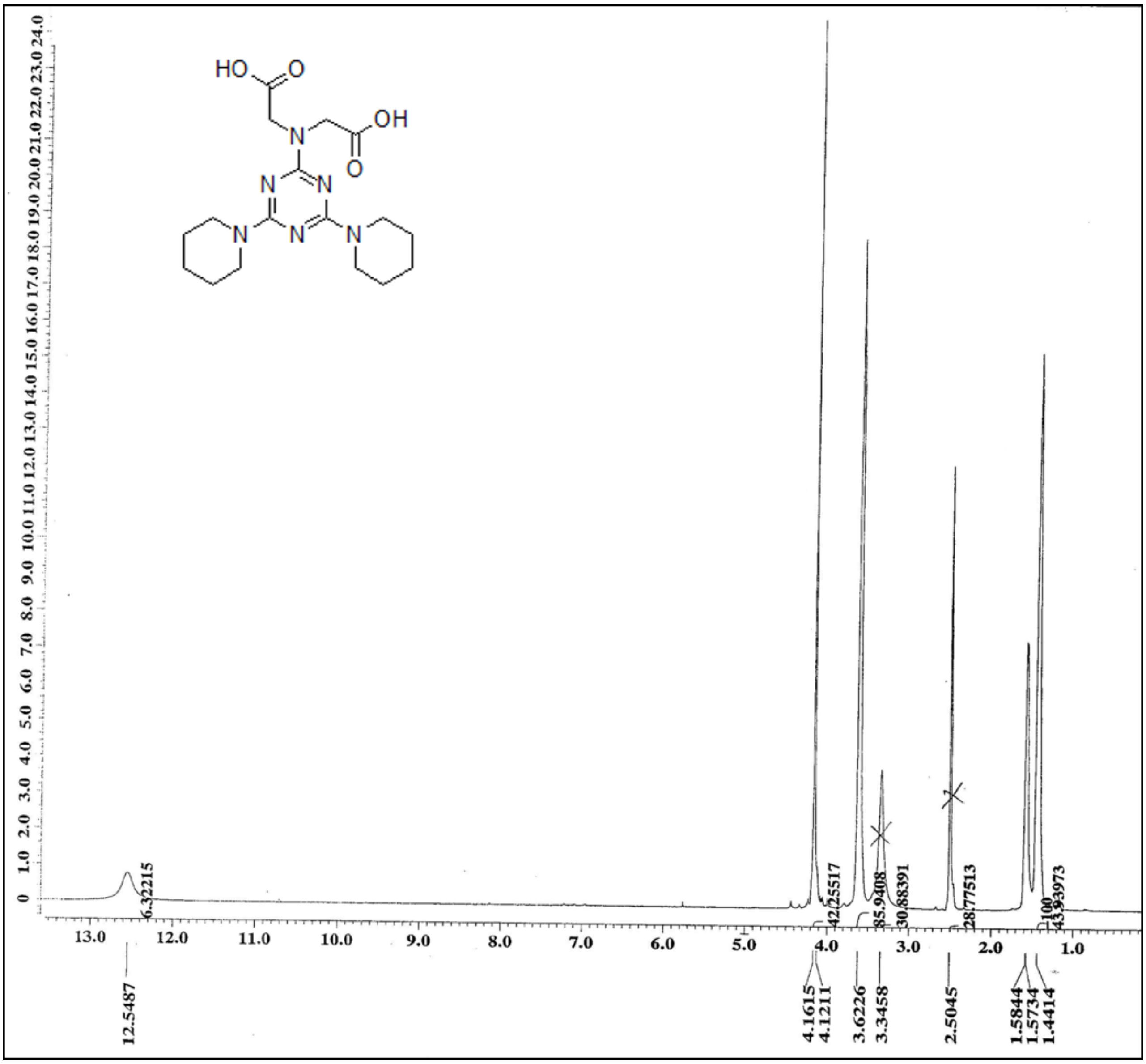

Figure S15.¹H-NMR (400 MHz, DMSO-d_6) spectra of *N*-(4,6-dipiperidino-1,3,5-triazin-2-yl) iminodiacetic acid **15**.

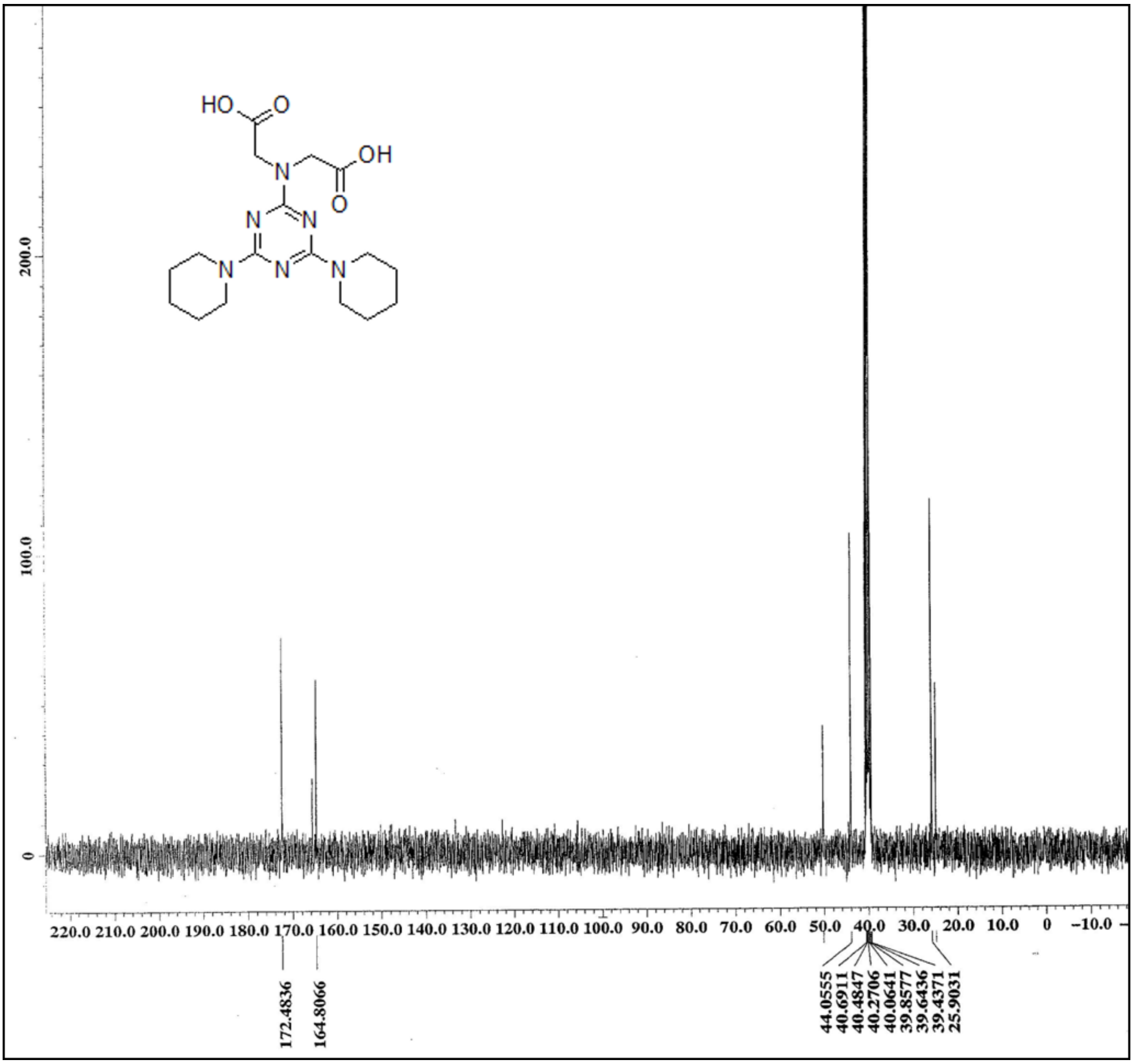

Figure S16. ^{13}C-NMR (400 MHz, DMSO-d_6) spectra of *N*-(4,6-dipiperidino-1,3,5-triazin-2-yl)iminodiacetic acid **15**.

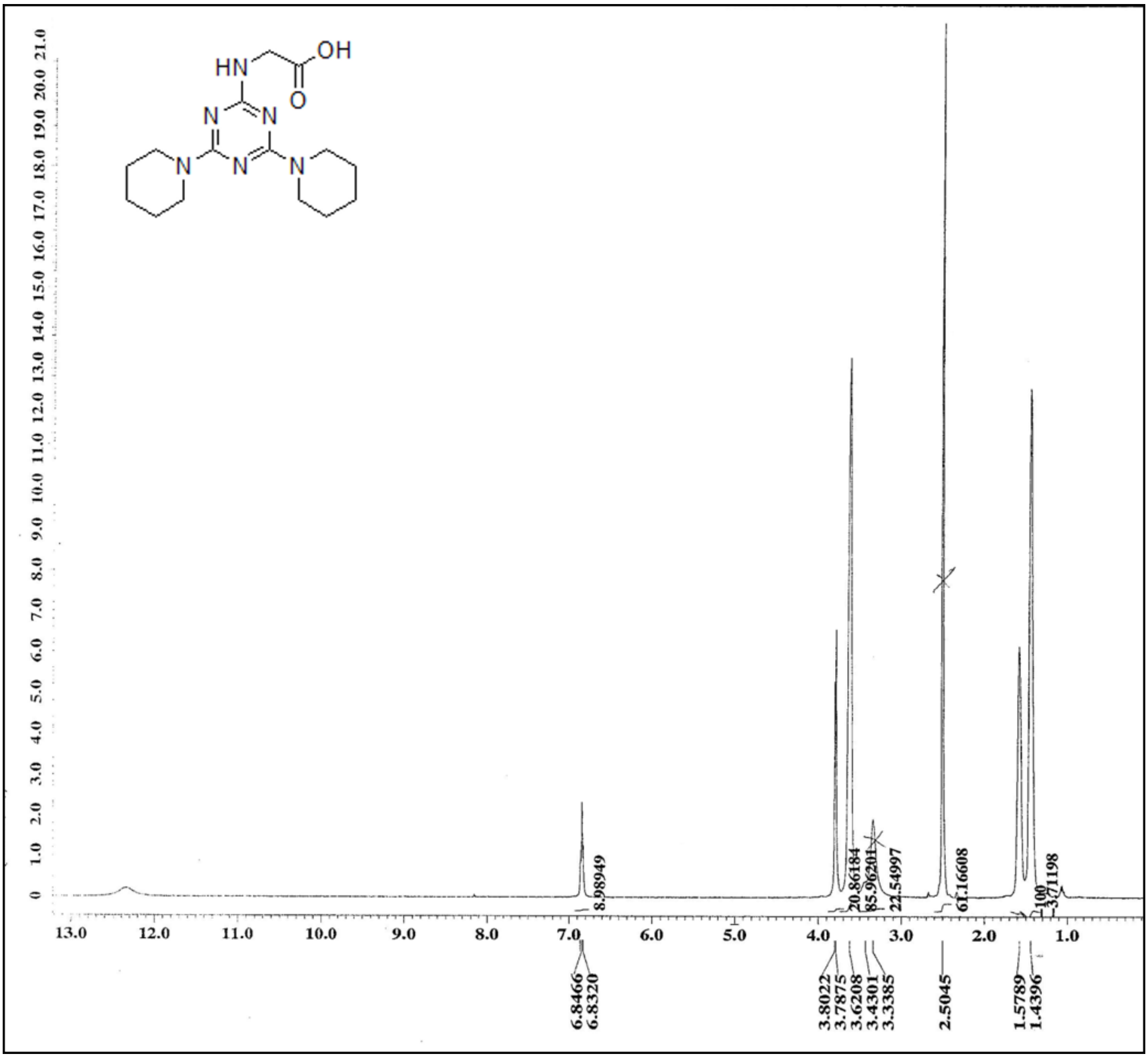

Figure S17. ^{1}H-NMR (DMSO-d_6) spectra of 2-(4,6-di(piperidin-1-yl)-1,3,5-triazin-2-ylamino) acetic acid **16**.

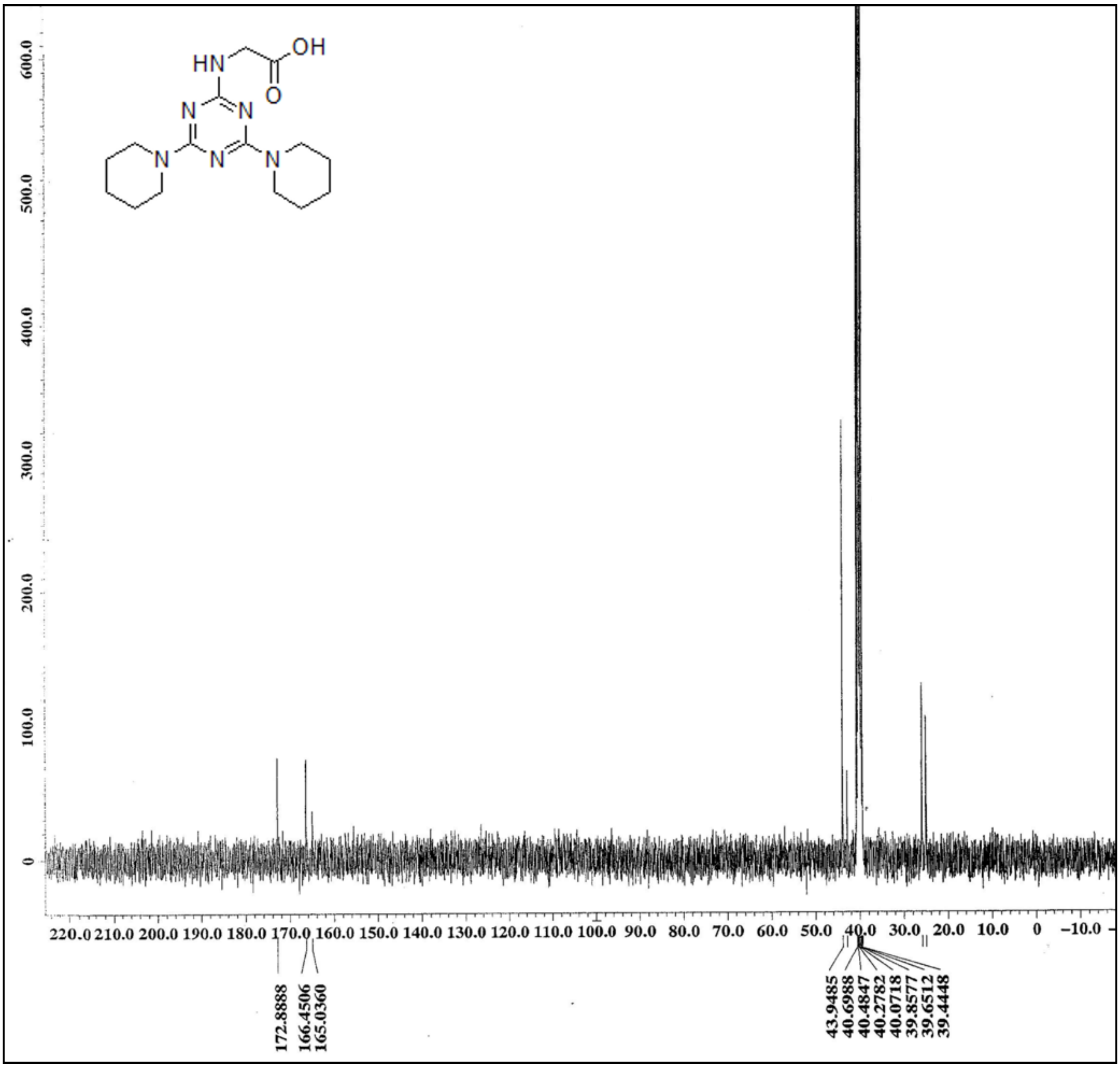

Figure S18. ^{13}C-NMR (DMSO-d_6) spectra of 2-(4,6-di(piperidin-1-yl)-1,3,5-triazin-2-ylamino) acetic acid **16**.

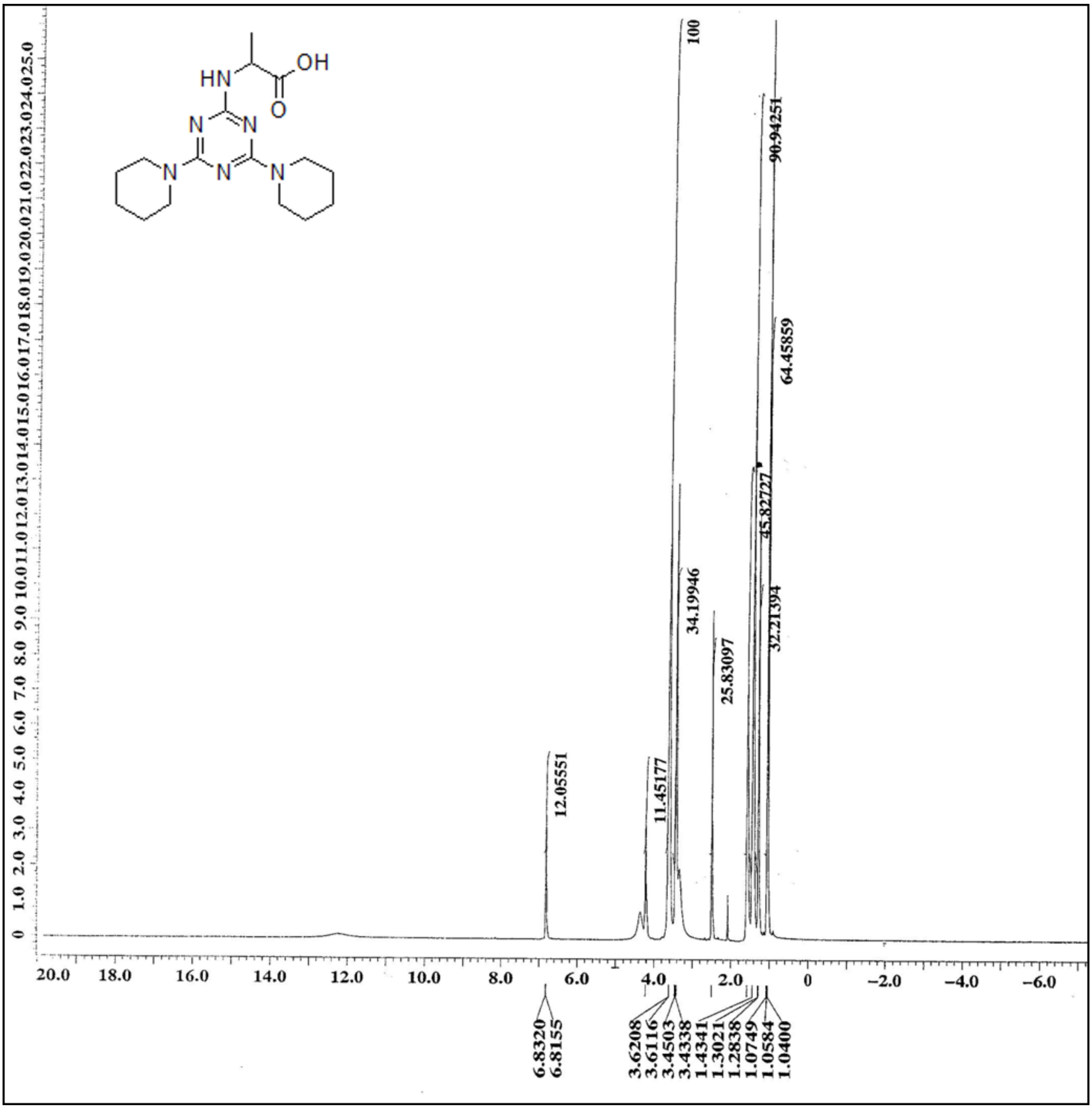

Figure S19. ^{1}H-NMR (DMSO-d_6) spectra of 2-(4,6-di(piperidin-1-yl)-1,3,5-triazin-2-ylamino) propanoic acid **17**.

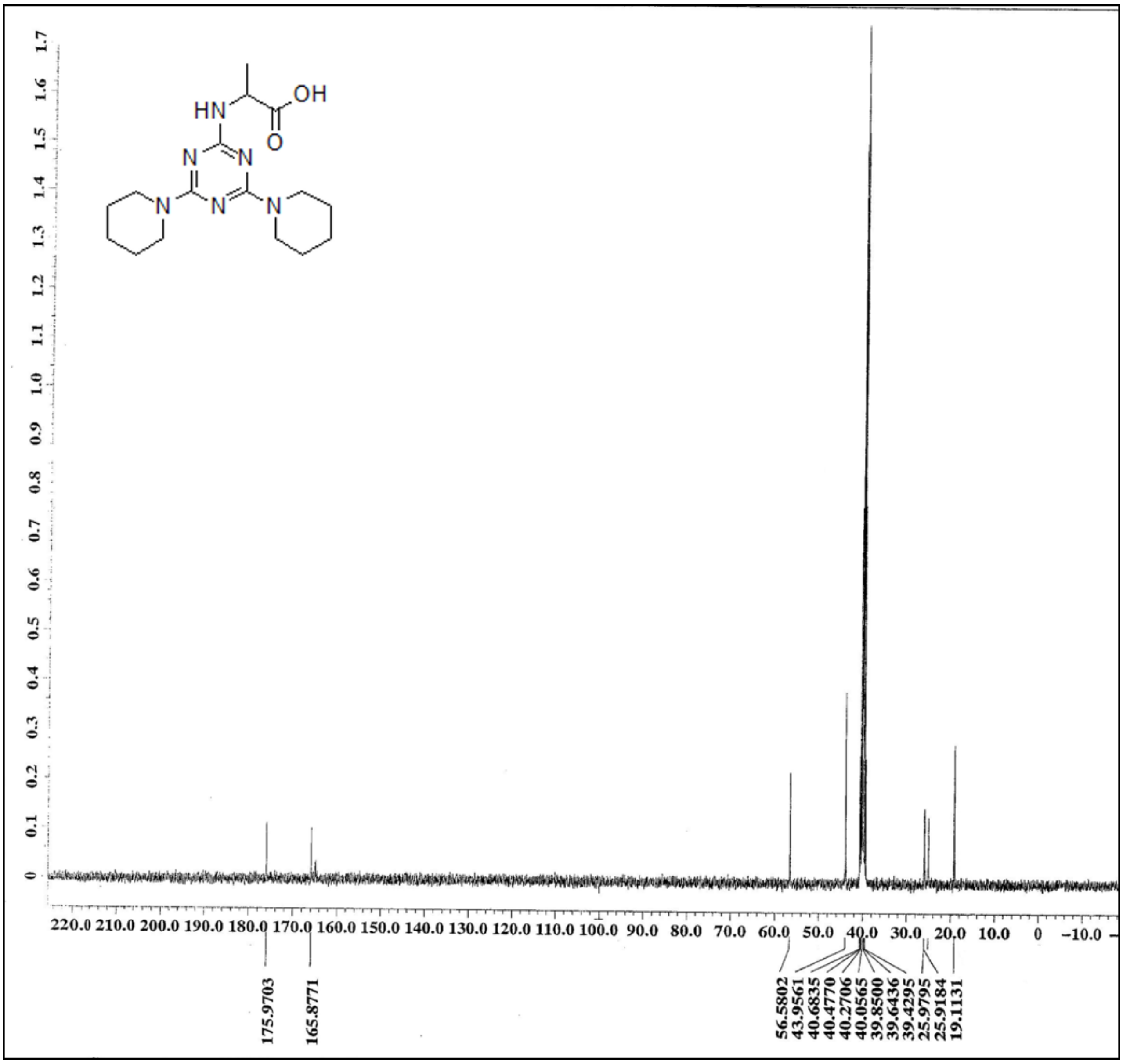

Figure S20. ^{13}C-NMR (DMSO-d_6) spectra of 2-(4,6-di(piperidin-1-yl)-1,3,5-triazin-2-ylamino) propanoic acid **17**.

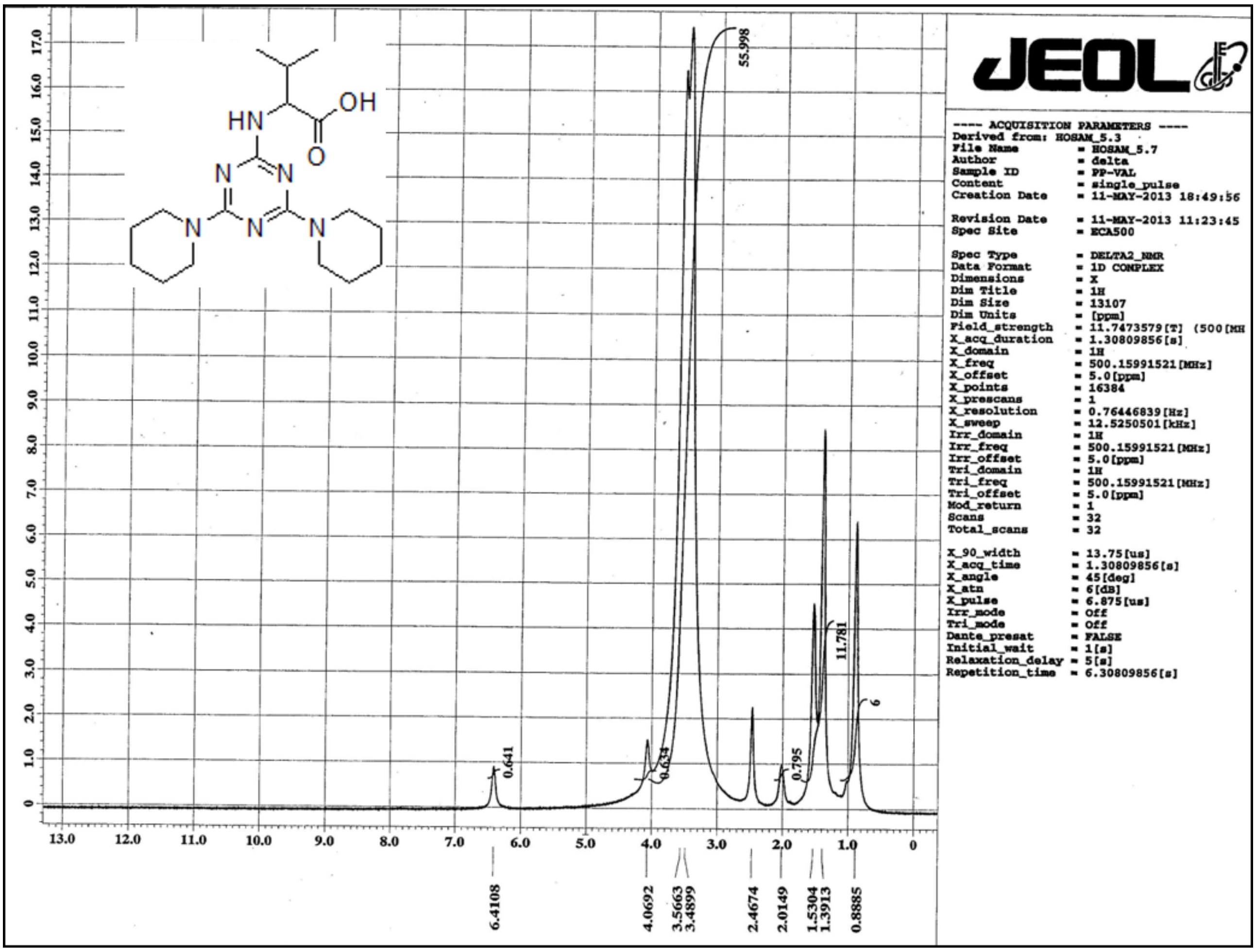

Figure S21.^{1}H-NMR (500 MHz, DMSO-d_6) spectrum of 2-(4,6-di(piperidin-1-yl)-1,3,5-triazin-2-ylamino)-3-methylbutanoic acid **18**.

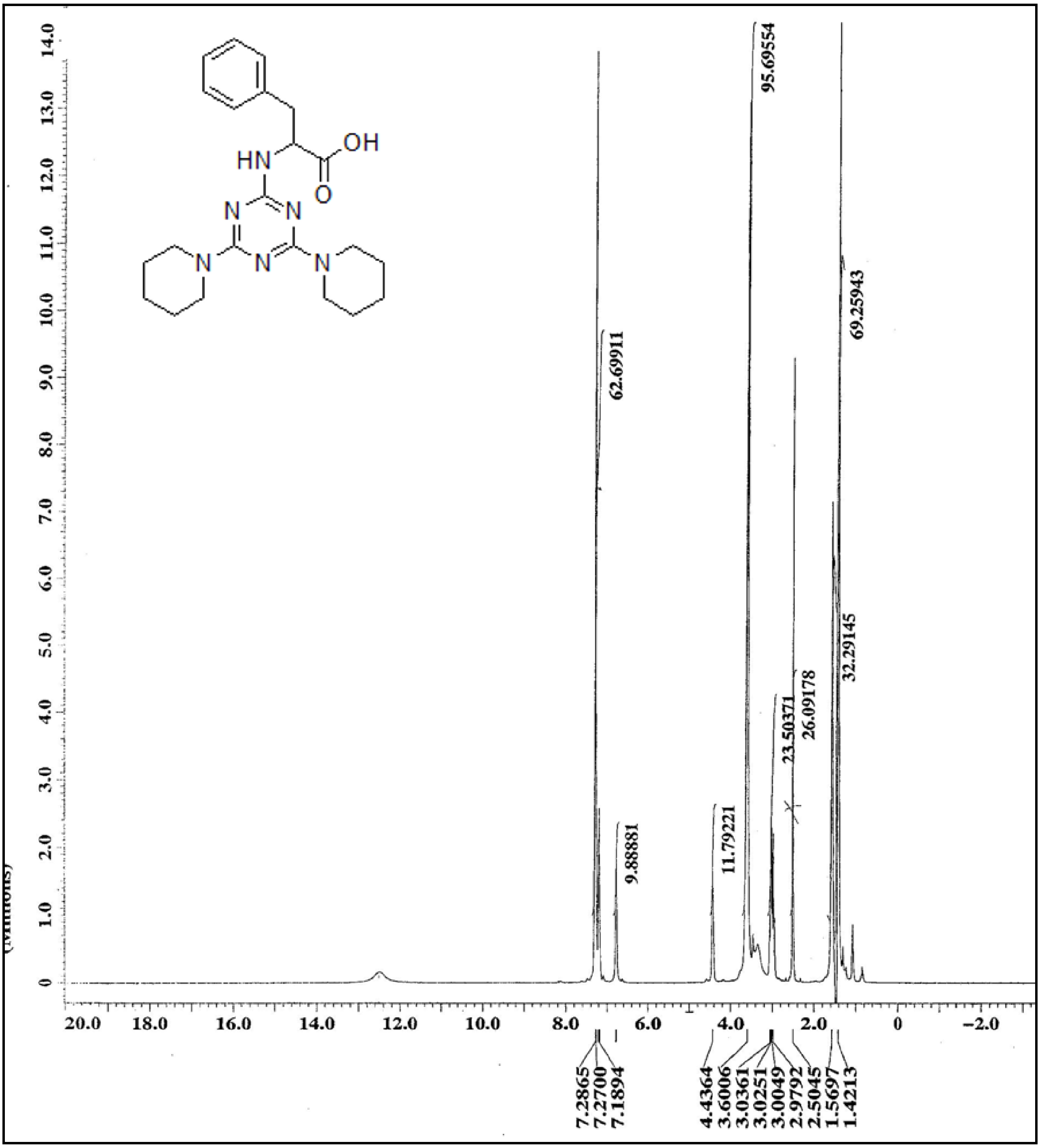

Figure S22. ^{1}H-NMR (DMSO-d_6) spectra of 2-(4,6-di(piperidin-1-yl)-1,3,5-triazin-2-ylamino)-3-phenylpropanoic acid **19**.

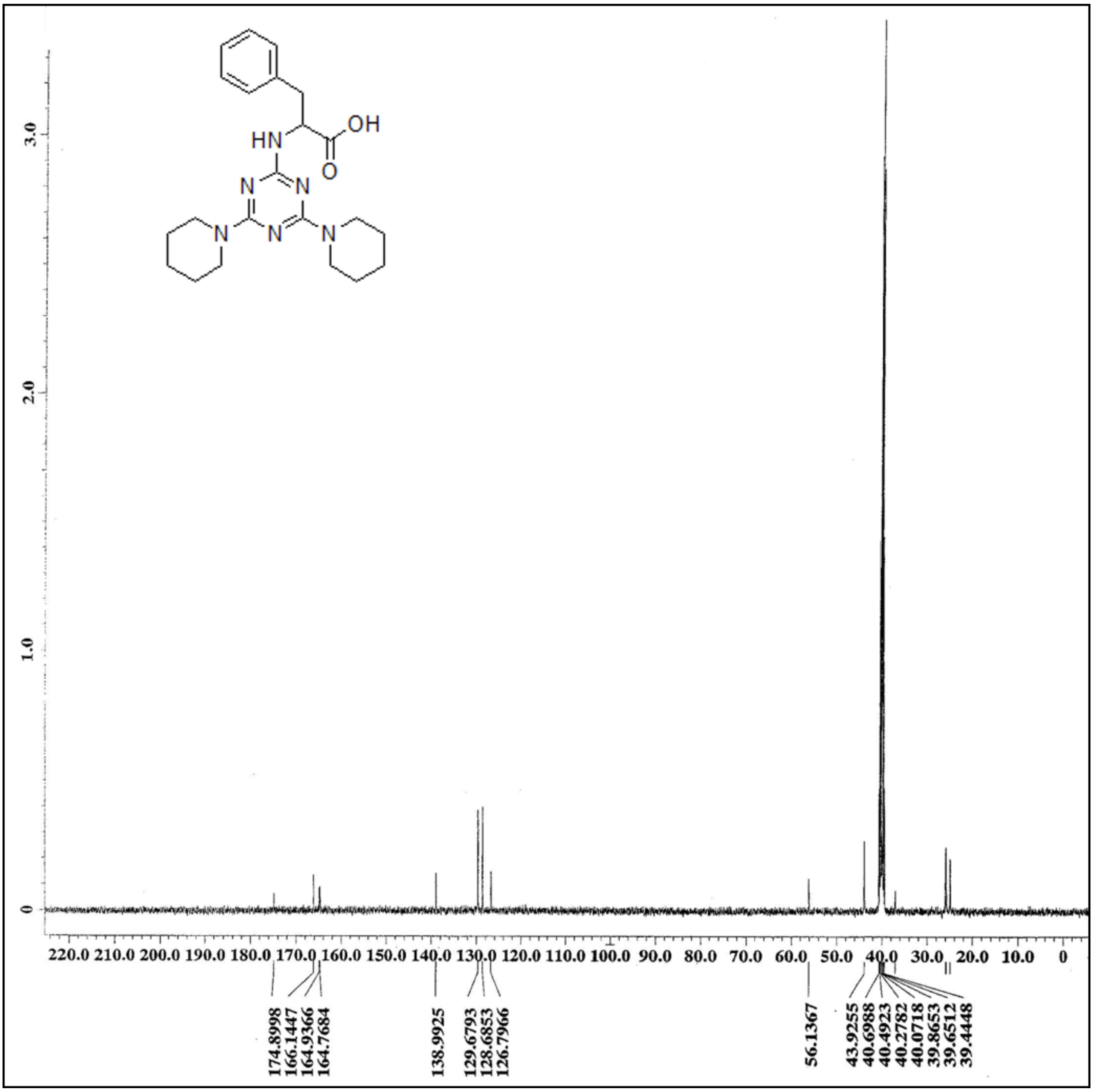

Figure S23. ^{13}C-NMR (DMSO-d_6) spectra of 2-(4,6-di(piperidin-1-yl)-1,3,5-triazin-2-ylamino)-3-phenylpropanoic acid **19**.

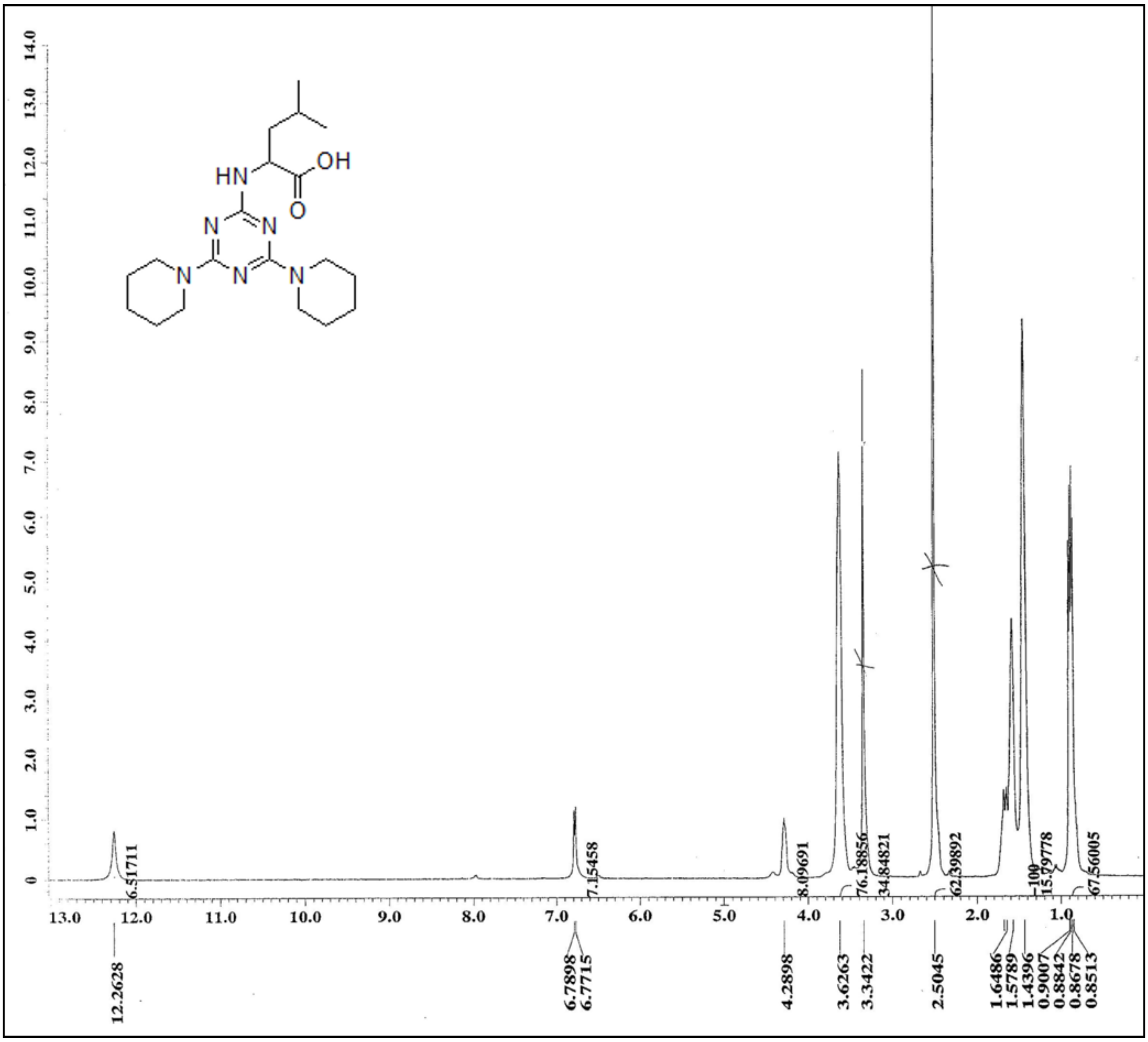

Figure S24. ^{1}H-NMR (DMSO-d_6) spectra of 2-(4,6-di(piperidin-1-yl)-1,3,5-triazin-2-ylamino)-4-methylpentanoic acid **20**.

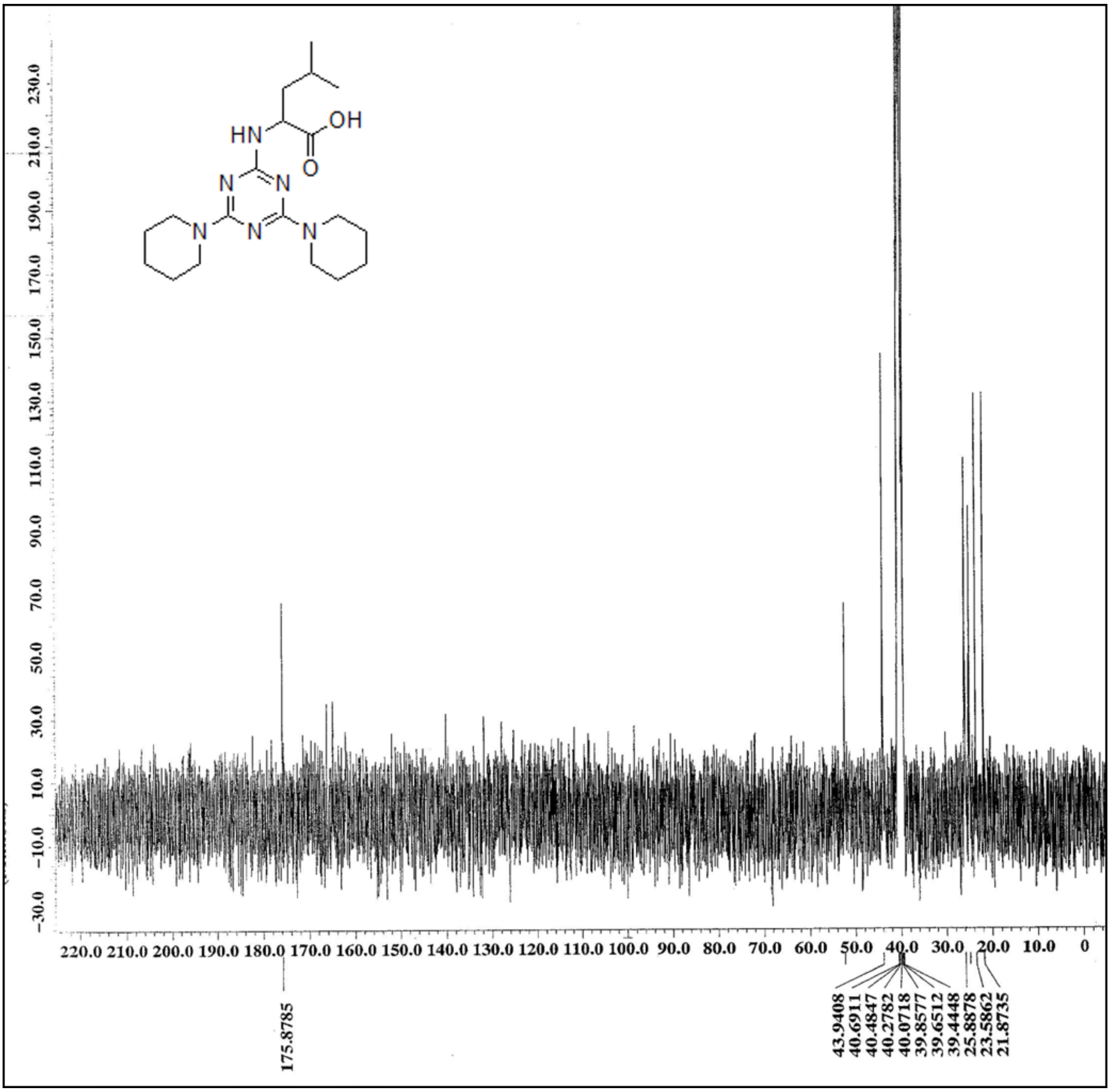

Figure S25. ^{13}C-NMR (DMSO-d_6) spectra of 2-(4,6-di(piperidin-1-yl)-1,3,5-triazin-2-ylamino)-4-methylpentanoic acid **20**.

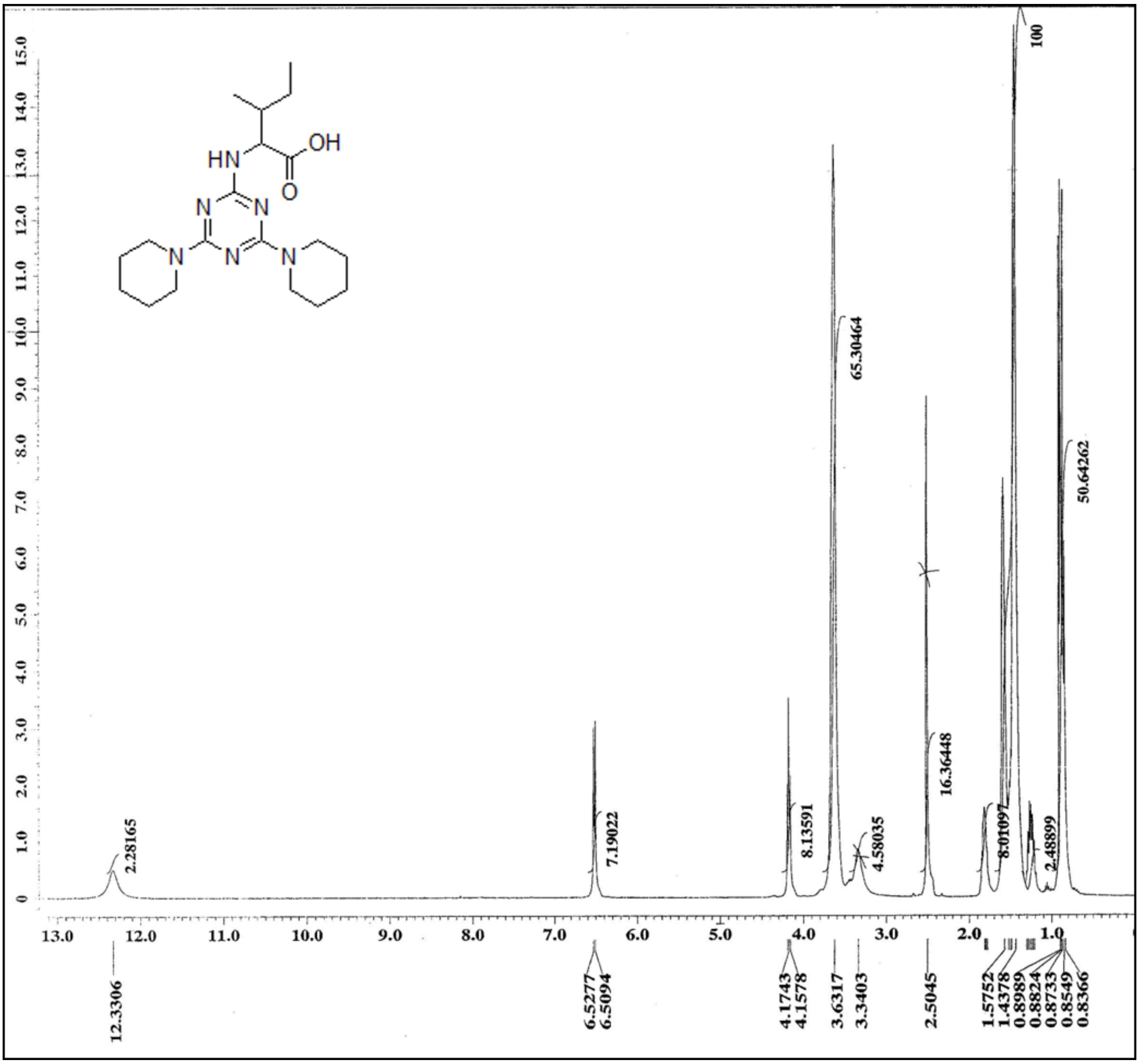

Figure S26. ^{1}H-NMR (DMSO-d_6) spectra of 2-(4,6-di(piperidin-1-yl)-1,3,5-triazin-2-ylamino)-3-methylpentanoic acid **21**.

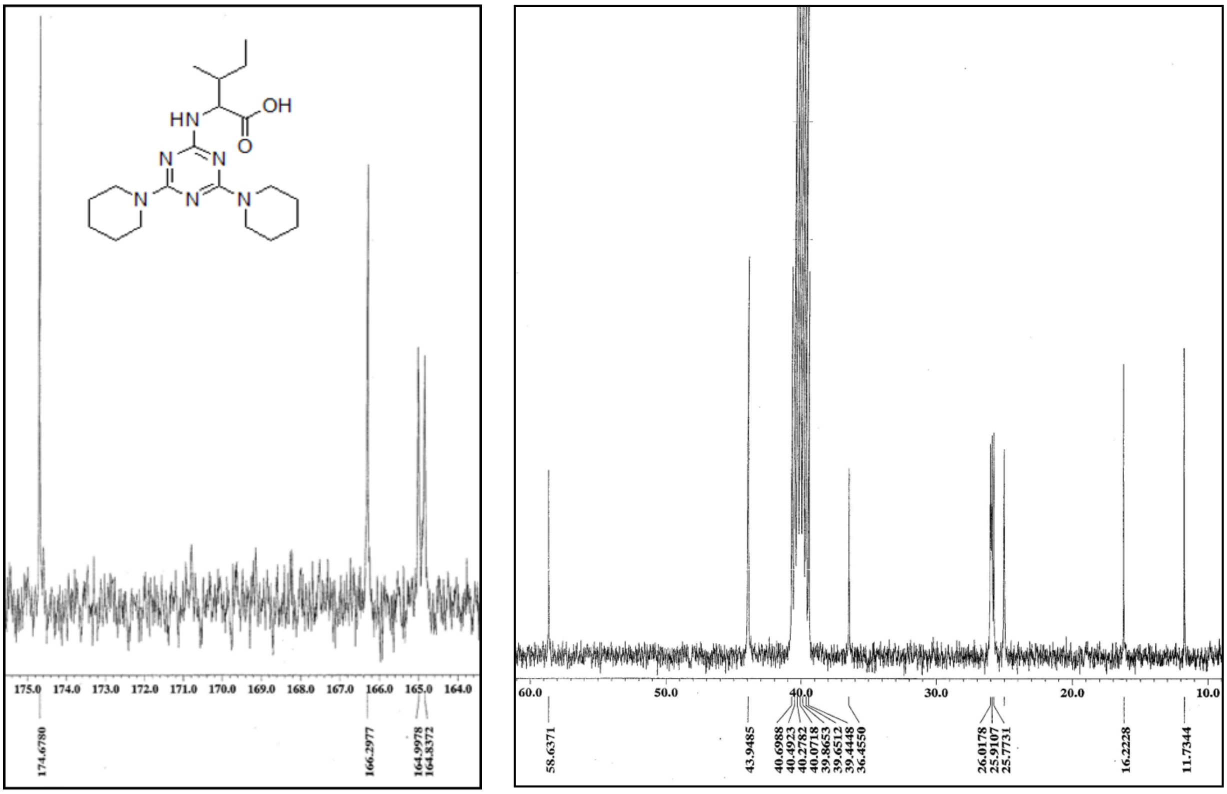

Figure S27. ^{13}C-NMR (DMSO-d_6) spectra of 2-(4,6-di(piperidin-1-yl)-1,3,5-triazin-2-ylamino)-3-methylpentanoic acid **21**.

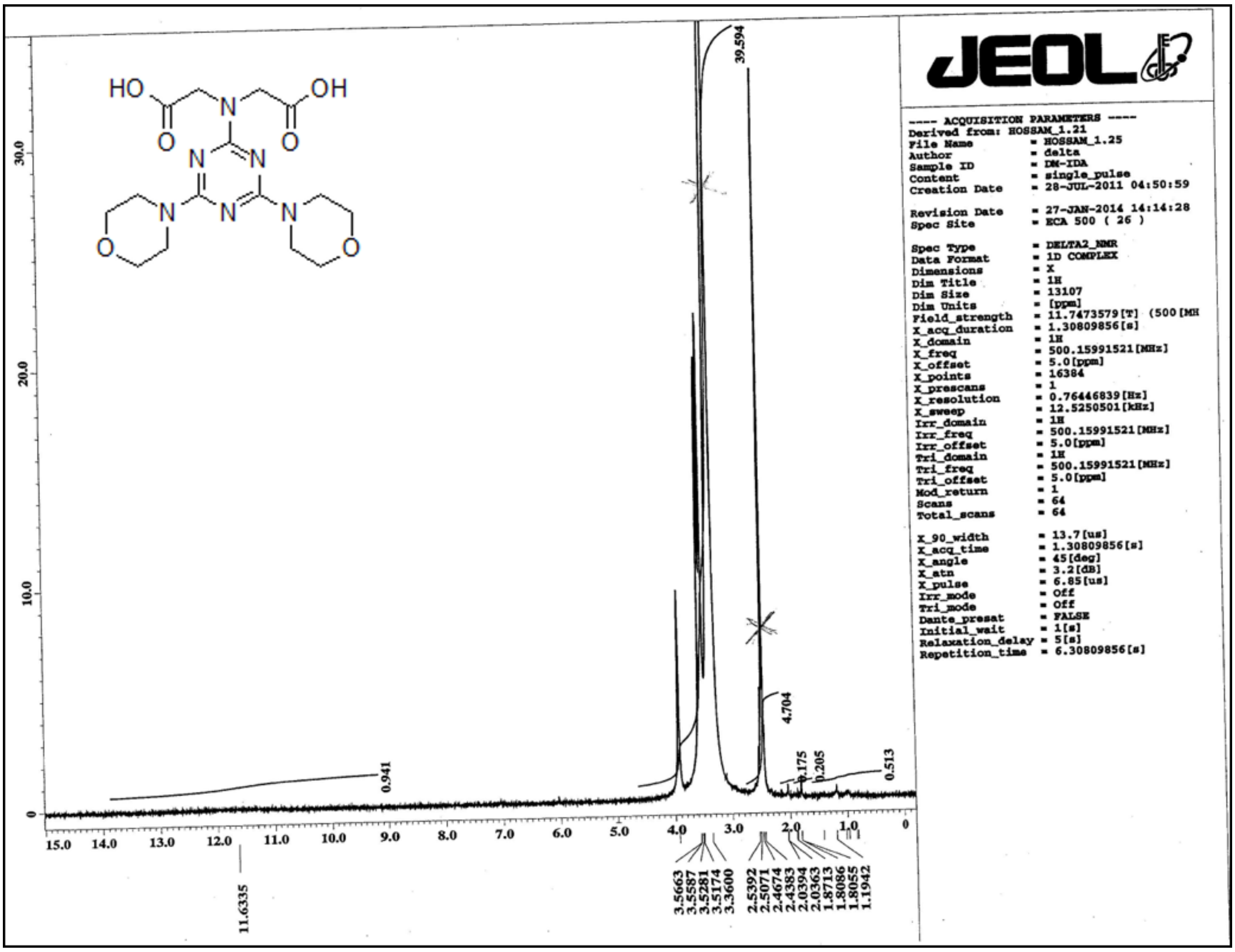

Figure S28. ^{1}H-NMR (500 MHz, DMSO-d_6) spectrum of *N*-(4,6-dimorpholino-1,3,5-triazin-2-yl)iminodiacetic acid **22**.

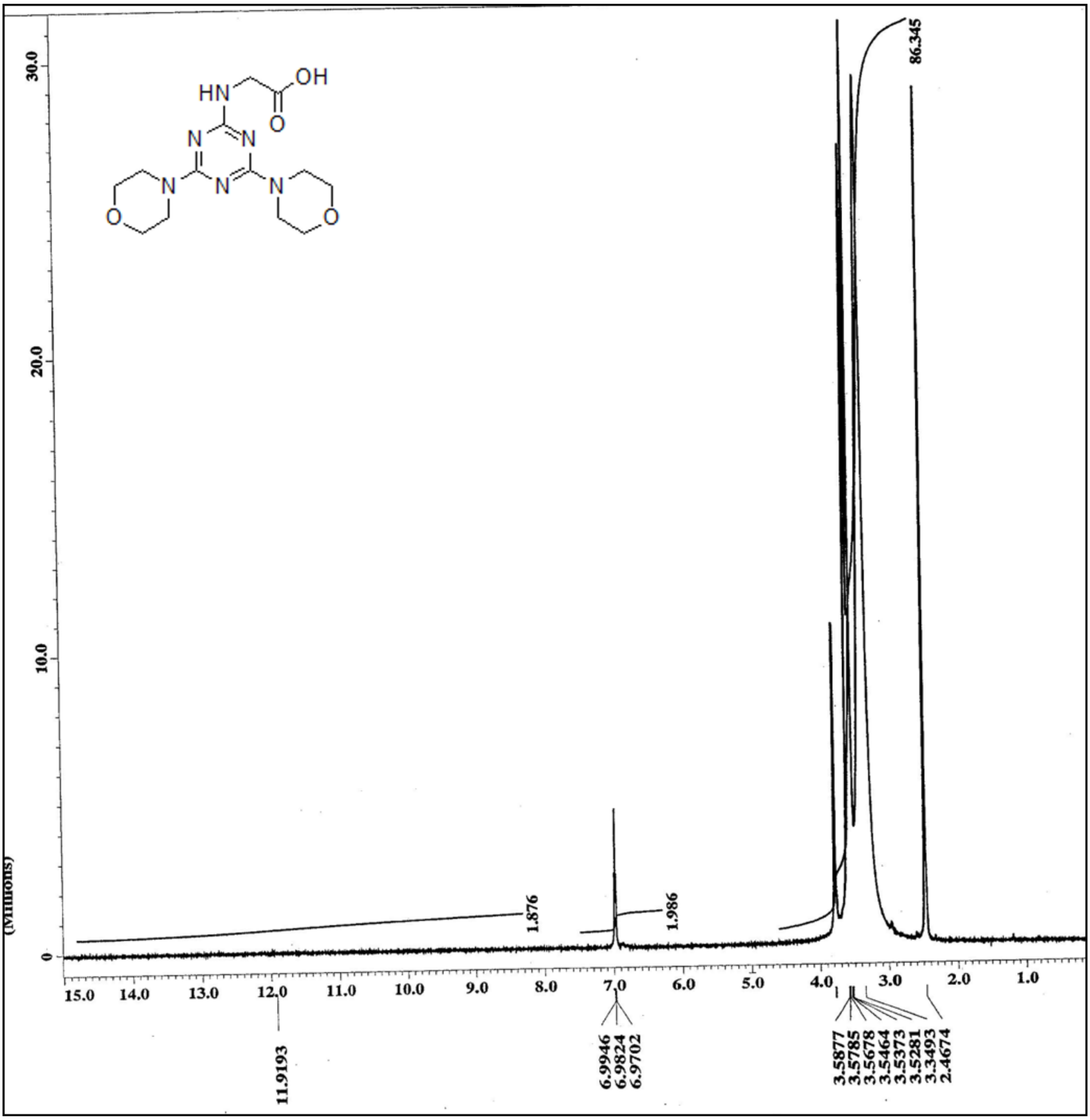

Figure S29. ^{1}H-NMR (DMSO-d_6) spectra of 2-(4,6-dimorpholino-1,3,5-triazin-2-ylamino) acetic acid **23**.

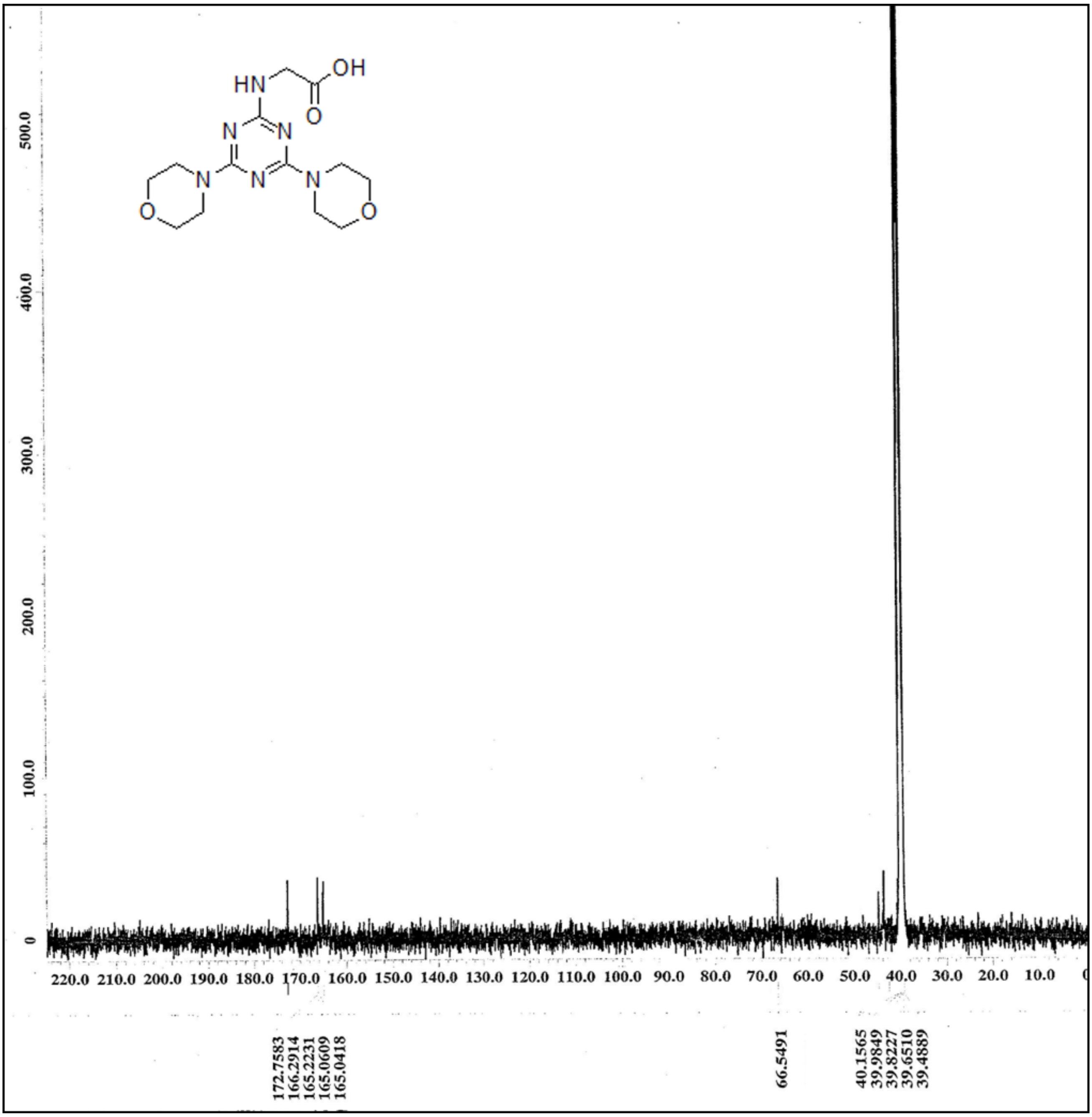

Figure S20. ^{13}C-NMR (DMSO-d_6) spectra of 2-(4,6-dimorpholino-1,3,5-triazin-2-ylamino) acetic acid **23**.

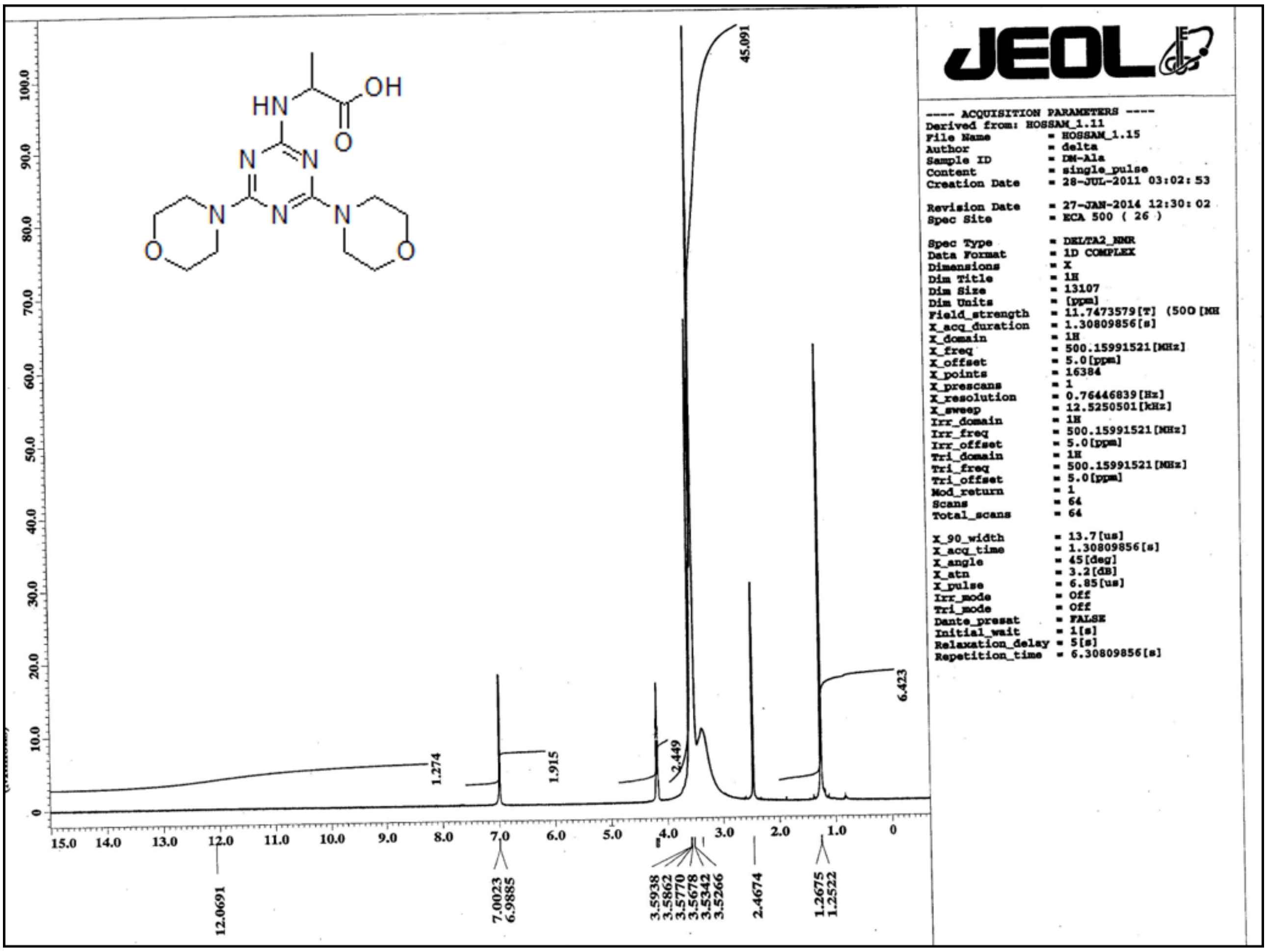

Figure S31. ^{1}H-NMR (500 MHz, DMSO-d_6) spectrum of 2-(4,6-dimorpholino-1,3,5-triazin-2-ylamino)propanoic acid **24**.

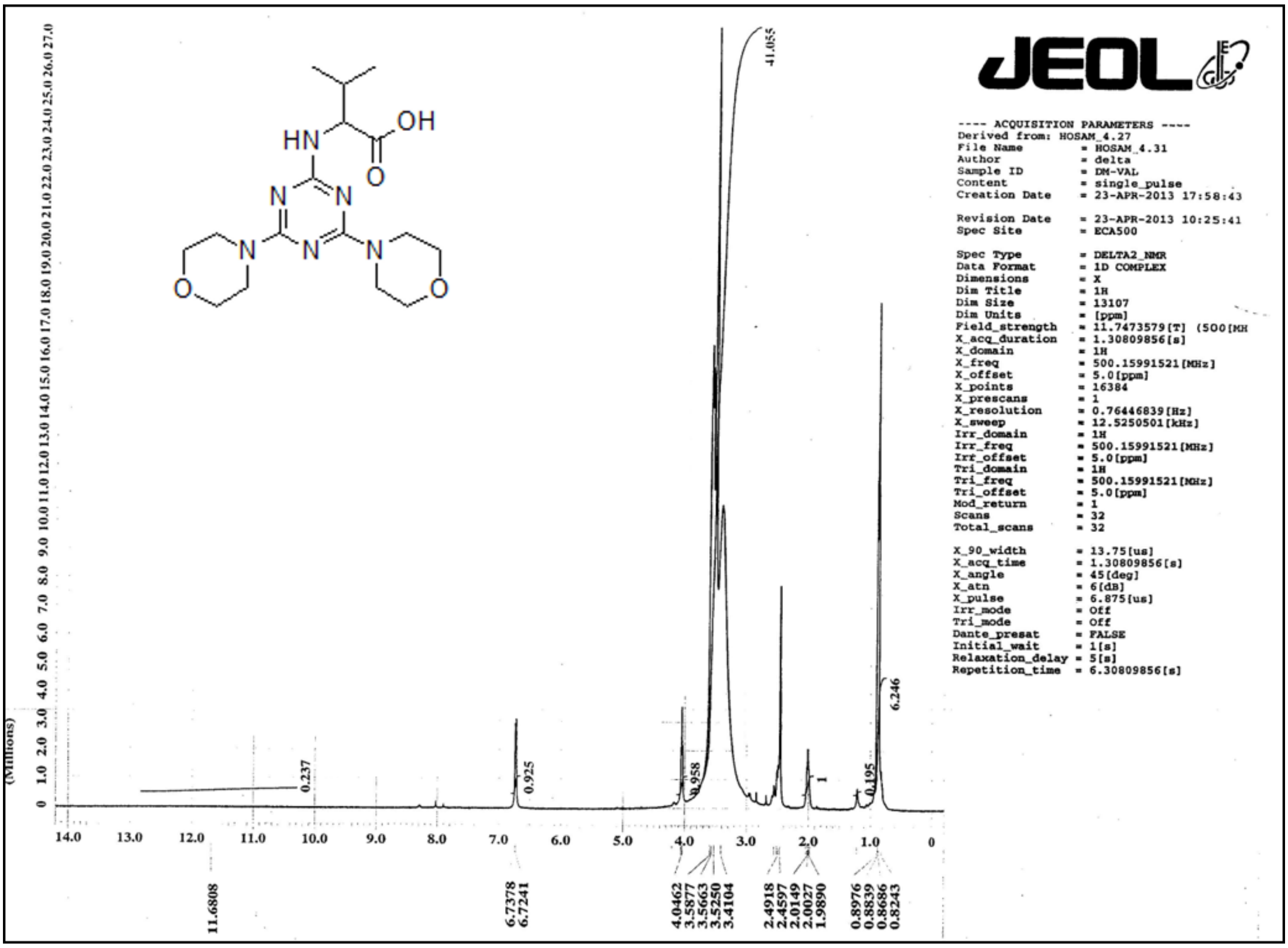

Figure S32. ^{1}H-NMR (500 MHz, DMSO-d_6) spectrum of 2-(4,6-dimorpholino-1,3,5-triazin-2-ylamino)-3-methylbutanoic acid **25**.

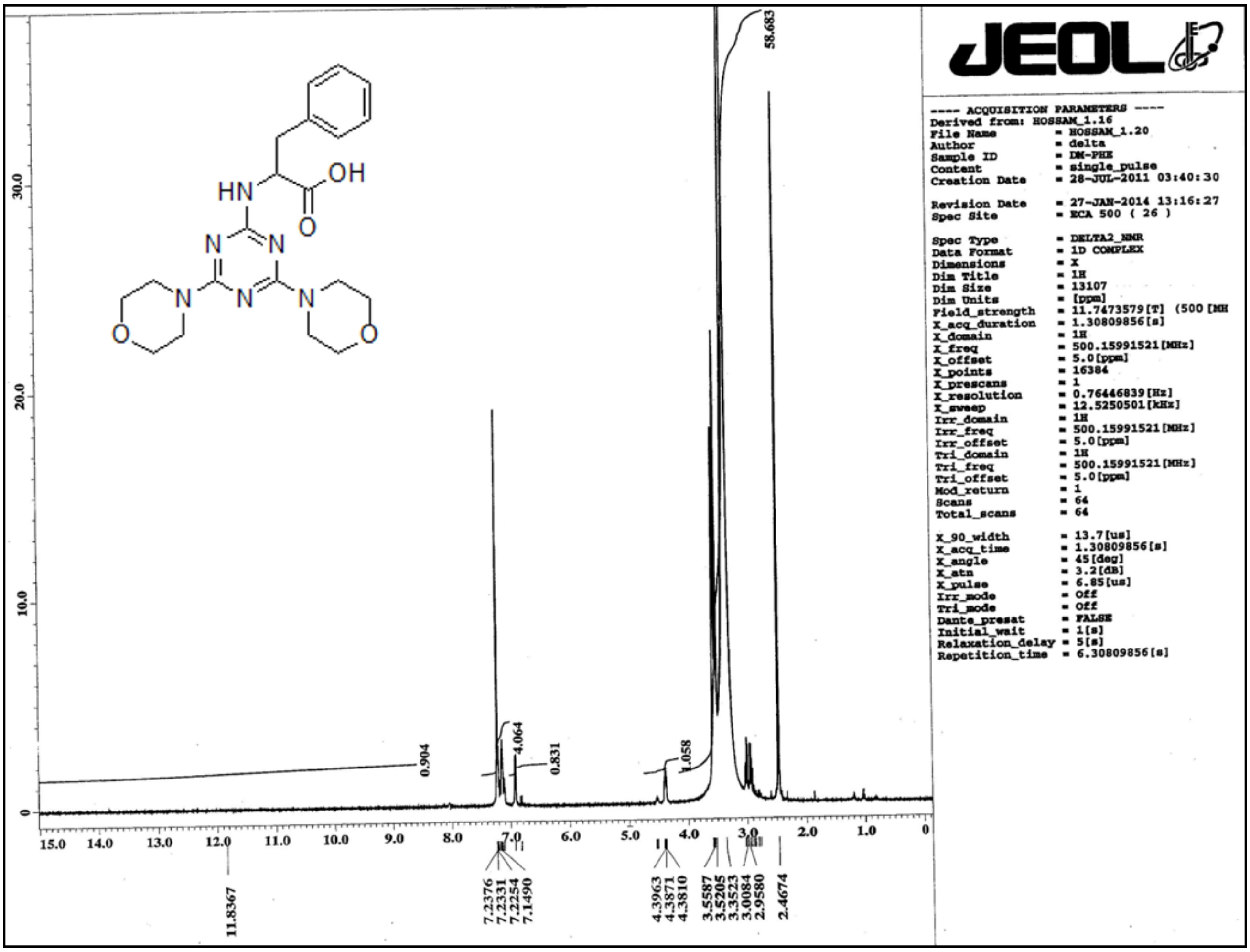

Figure S33. ^{1}H-NMR (500 MHz, DMSO-d_6) spectrum of 2-(4,6-dimorpholino-1,3,5-triazin-2-ylamino)-3-phenylpropanoic acid **26**.

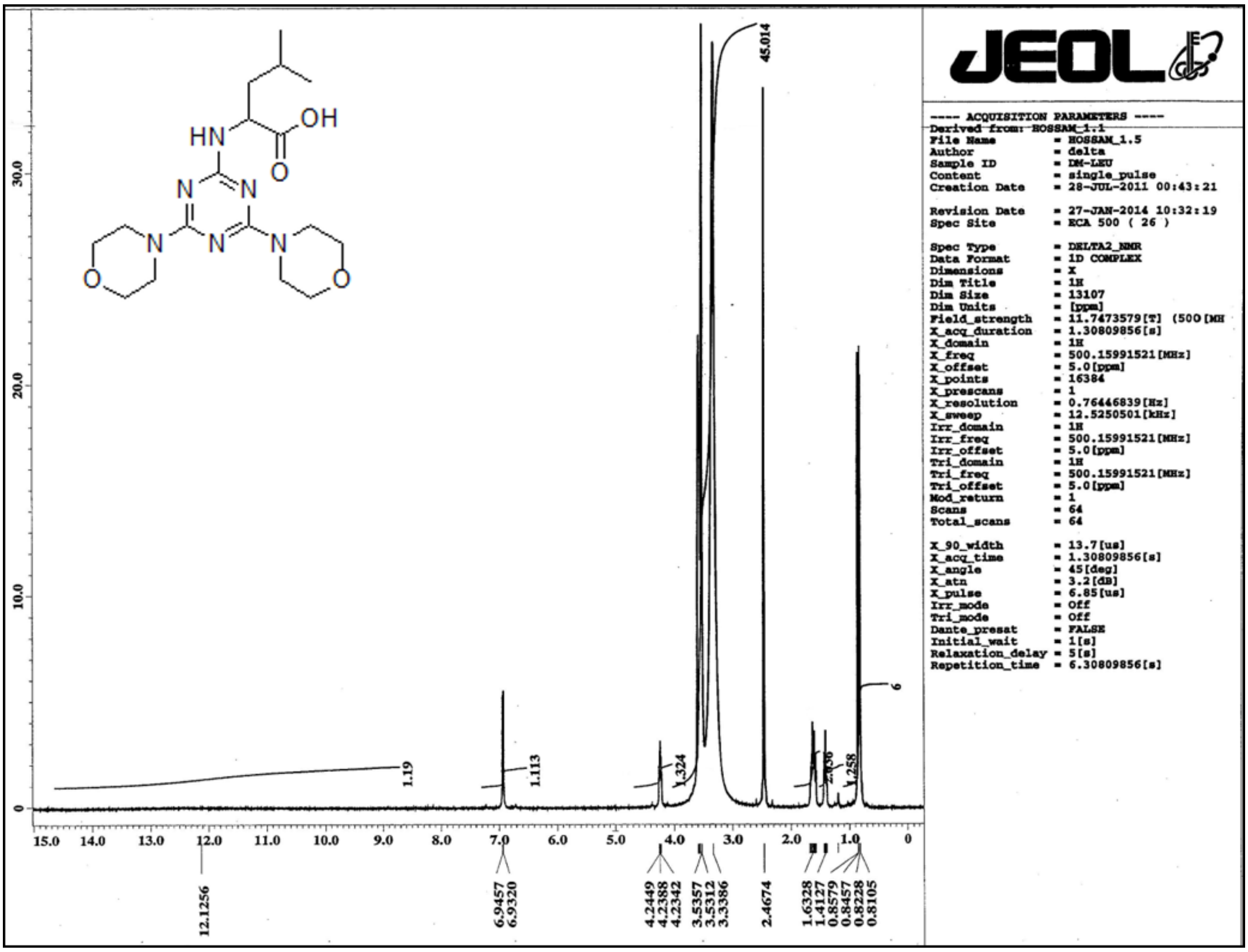

Figure S34. ^{1}H-NMR (500 MHz, DMSO-d_6) spectrum of 2-(4,6-dimorpholino-1,3,5-triazin-2-ylamino)-4-methylpentanoic acid **27**.

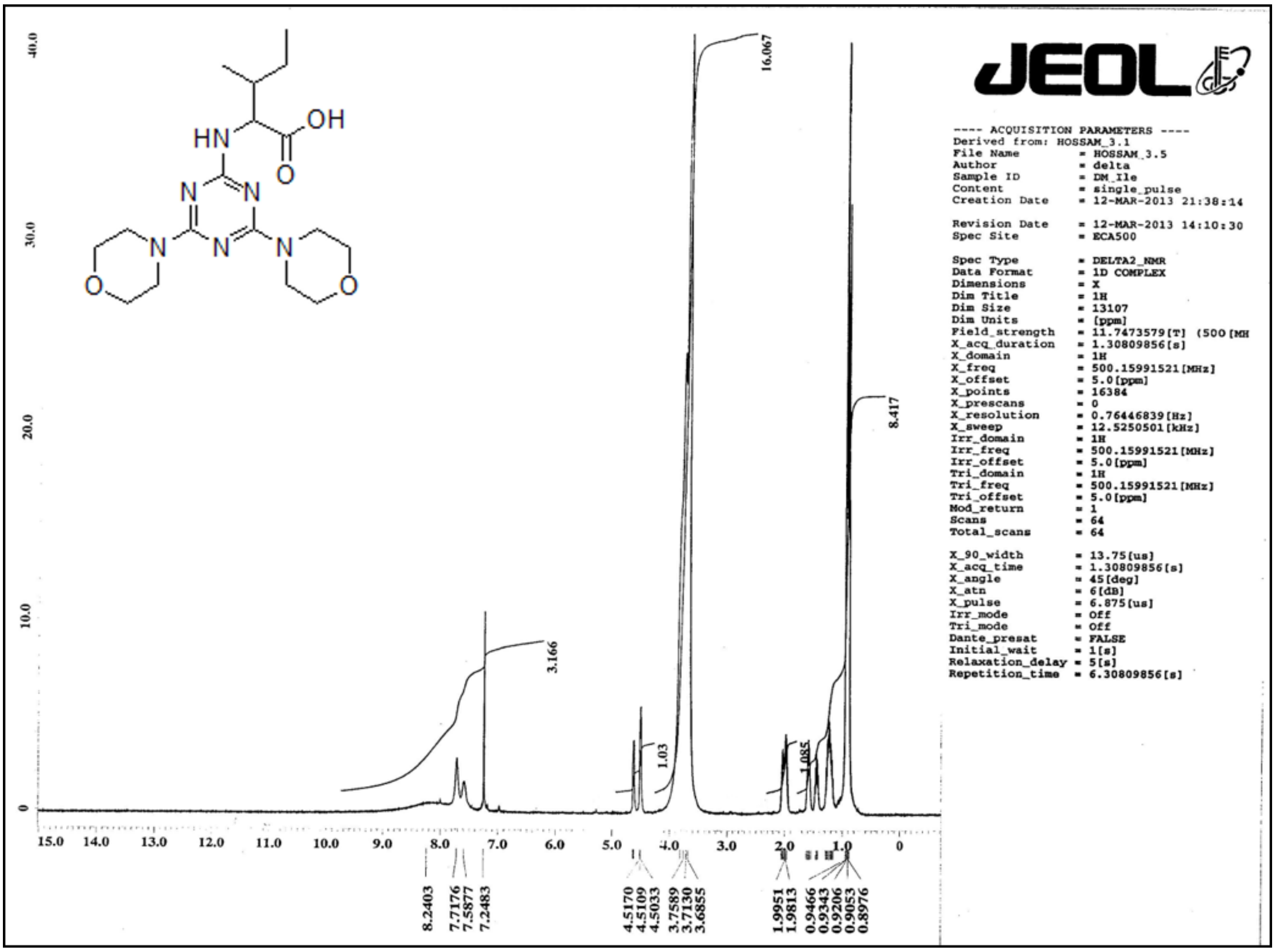

Figure S35. ^{1}H-NMR (500 MHz, $CDCl_3$) spectrum of 2-(4,6-dimorpholino-1,3,5-triazin-2-ylamino)-3-methylpentanoic acid **28**.

Preliminary Biological Tests

The newly synthesized compounds **3–9** and **15–28** were tested to determine their activity toward MAO-A and MAO-B selectivity in the presence of the specific substrate, serotonin or benzylamine (l00 μM), respectively. Bovine brain mitochondria were isolated according to Basford [1]. The activities of MAO-A and MAO-B were determined using a fluorimetric method described by Matsumoto *et al.* [2]. The mitochondrial fractions were incubated at 38 °C for 30 min with the specific inhibitor of one of MAO isoforms, L-deprenyl (l0.5 μM) to determine MAO-A activity or clorgyline (10.5 μM) to determine MAO-B activity. The activity of MAO-A or MAO-B was determined using the corresponding mitochondrial fraction previously treated andcontaining one active enzyme, incubationmixture contained 0.1 mL phosphate buffer (0.25 M, pH 7.4), mitochondrial suspension (6 mg/L, the specific substrate for MAO-A or MAO-B (0.1 μM) and test compounds at five different concentrations ranging from 0.5 nM to 0.1 mM (0, 0.5 nM, 5 nM, 5 μM, 50 μM and 100 μM) were dissolved in propylene glycol. The mixture was incubated in a shaking water-bath at 37 °C for 60 min. The reaction was quenched by adding perchloric acid. The samples were centrifuged at 10,000× *g* for 5 min and the supernatant was completed to 2.7 mL using 1 N NaOH and measured with a Perkin-Elmer Lf 45 Spectrofluorimeter. The IC_{50}value was calculated and analyzed using the four-parameter logistic function in Sigma Plot software (SigmaPlot 12.3, Systat

Software. Inc., Richmond, CA, USA). Protein concentration was determined according to a previously reported method [3]. The MAO-A and MAO-Bresults are expressed as IC_{50} (Table 1). Propylene glycol was used as negative control and did not show any effect on the enzyme activity.

Table 1. Effect of 2,4-disubstituted-1,3,5-triazine-based amino acid derivatives on MAO-A and MAO-B activity.

Compound	IC_{50} (M)		Selectivity Index (SI) [a]
	MAO-A	MAO-B	
**3**	$5.8{\times}10^{-8} \pm 0.13$	$3.9 \times 10^{-4} \pm 0.28$	6724
**4**	$8.4{\times}10^{-8} \pm 0.12$	$9.8 \times 10^{-4} \pm 0.32$	11667
**5**	$6.4 \times 10^{-8} \pm 0.14$	$9.4 \times 10^{-4} \pm 0.18$	14688
**6**	$5.1 \times 10^{-9} \pm 0.48$	$4.4 \times 10^{-4} \pm 0.22$	68275
**7**	$3.1 \times 10^{-9} \pm 0.24$	$7.4 \times 10^{-4} \pm 0.28$	238710
**8**	$6.7 \times 10^{-8} \pm 0.44$	$5.8 \times 10^{-4} \pm 0.12$	8657
**9**	$7.4 \times 10^{-8} \pm 0.22$	$4.8 \times 10^{-4} \pm 0.11$	6486
**15**	$8.8 \times 10^{-8} \pm 0.28$	$7.4 \times 10^{-4} \pm 0.24$	8409
**16**	$8.7 \times 10^{-8} \pm 0.13$	$7.4 \times 10^{-4} \pm 0.34$	8506
**17**	$6.1 \times 10^{-8} \pm 0.26$	$4.4 \times 10^{-4} \pm 0.46$	7213
**18**	$3.2 \times 10^{-9} \pm 0.22$	$5.4 \times 10^{-4} \pm 0.16$	168750
**19**	$7.8 \times 10^{-9} \pm 0.28$	$5.5 \times 10^{-4} \pm 0.18$	70513
**20**	$3.2 \times 10^{-8} \pm 0.22$	$9.8 \times 10^{-4} \pm 0.14$	30625
**21**	$2.8{\times}10^{-8} \pm 0.18$	$8.2 \times 10^{-4} \pm 0.27$	29286
**22**	$7.2 \times 10^{-8} \pm 0.36$	$6.1 \times 10^{-4} \pm 0.32$	8472
**23**	$5.8 \times 10^{-8} \pm 0.38$	$4.8 \times 10^{-4} \pm 0.28$	8276
**24**	$6.4 \times 10^{-8} \pm 0.14$	$5.7 \times 10^{-4} \pm 0.10$	8906
**25**	$3.5 \times 10^{-9} \pm 0.28$	$8.6 \times 10^{-4} \pm 0.26$	245714
**26**	$4.2 \times 10^{-8} \pm 0.32$	$7.8 \times 10^{-4} \pm 0.28$	18571
**27**	$2.9 \times 10^{-8} \pm 0.12$	$8.8 \times 10^{-4} \pm 0.14$	30345
**28**	$3.2 \times 10^{-8} \pm 0.16$	$9.2 \times 10^{-4} \pm 0.22$	28750
**Clorgyline**	$2.9 \times 10^{-9} \pm 0.12$	$9.8 \times 10^{-5} \pm 0.16$	33793

The results were expressed as mean ± S.E.M. Data were analyzed by one-way of variance. Student's test for unpaired observations was used. p-value ≤ 0.001 and was significant. The number of experiments was 6.
[a] SI = MAO-B IC_{50}/MAO-A IC_{50}.

Oral acute toxicity of the test compounds was studied using male mice (20 g each, Medical Research Institute, Alexandria University) according to previously reported methods [4]. The animals were divided into groups of six mice each. The compounds were given orally, suspended in 1% gum acacia, in doses of 50, 150, 200, 250 mg/kg. The mortality percentage in each group was recorded after 24 h. Additionally, the test compounds were investigated for their parenteral acute toxicity [5] in groups of mice of six animals each. The compounds or their vehicle, propylene glycol (control), were given by intraperitoneal injection in doses of 25, 50, 75, 100, 125 mg/kg. The percentage survival was followed up to 7 days.

References

1. Basford, R.E. Preparation and properties of brain mitochondria. *Methods Enzymol.* **1967**, *10*, 96–101.
2. Matsumoto, T.; Suzuki, O.; Furuta, T.; Asai, M.; Kurokawa, Y.; Rimura, Y.; Katsumata, Y.; Takahashi, I. A sensitive fluorometric assay for serum monoamine oxidase with kynuramine as substrate. *Clin. Biochem*. **1985**, *18*, 126–129.
3. Bradford, M.M. A rapid and sensitive method for the quantitation of microgram quantities of protein utilizing the principle of protein-dye binding. *Anal. Biochem.* **1976**, *72*, 248–254.
4. Verma, M.; Tripathi, M.; Saxena, A.K.; Shanker, K. Antiinflammatory activity of novel indole derivatives. *Eur. J. Med. Chem.* **1994**, *29*, 941–946.
5. Bekhit, A.A.; Fahmy, H.T.Y. Design and Synthesis of Some Substituted 1H-Pyrazolyl-oxazolidines or 1*H*-Pyrazolyl-thiazolidines as Anti-inflammatory-Antimicrobial Agents. *Arch. Pharm. Pharm. Med. Chem.***2003**, *33*6, 111–118.
